# *Leishmania amazonensis* Engages CD36 to Drive Parasitophorous Vacuole Maturation

**DOI:** 10.1371/journal.ppat.1005669

**Published:** 2016-06-09

**Authors:** Kendi Okuda, Mei Tong, Brian Dempsey, Kathryn J. Moore, Ricardo T. Gazzinelli, Neal Silverman

**Affiliations:** 1 Division of Infectious Diseases & Immunology, Department of Medicine, University of Massachusetts Medical School, Worcester, Massachusetts, United States of America; 2 Department of Medicine, New York University School of Medicine, Langone Medical Center, New York, New York, United States of America; University of São Paulo FMRP/USP, BRAZIL

## Abstract

*Leishmania* amastigotes manipulate the activity of macrophages to favor their own success. However, very little is known about the role of innate recognition and signaling triggered by amastigotes in this host-parasite interaction. In this work we developed a new infection model in adult *Drosophila* to take advantage of its superior genetic resources to identify novel host factors limiting *Leishmania amazonensis* infection. The model is based on the capacity of macrophage-like cells, plasmatocytes, to phagocytose and control the proliferation of parasites injected into adult flies. Using this model, we screened a collection of RNAi-expressing flies for anti-*Leishmania* defense factors. Notably, we found three CD36-like scavenger receptors that were important for defending against *Leishmania* infection. Mechanistic studies in mouse macrophages showed that CD36 accumulates specifically at sites where the parasite contacts the parasitophorous vacuole membrane. Furthermore, CD36-deficient macrophages were defective in the formation of the large parasitophorous vacuole typical of *L*. *amazonensis* infection, a phenotype caused by inefficient fusion with late endosomes and/or lysosomes. These data identify an unprecedented role for CD36 in the biogenesis of the parasitophorous vacuole and further highlight the utility of *Drosophila* as a model system for dissecting innate immune responses to infection.

## Author Summary

Leishmaniasis is caused by *Leishmania* parasites and transmitted to humans by sandflies. After the establishment of infection, the intracellular parasite form, known as an amastigote, preferentially infects and replicates in macrophages, cells otherwise specialized for killing microbes. To overcome macrophage lethality, *Leishmania* possesses a sophisticated evasion strategy that sabotages macrophage defenses. The cell biology of *Leishmania*-macrophage interactions is not completely understood, because of the complexity of the host-parasite relationship and limited technical resources available in the classical mouse infection model. In this study we created a model of leishmaniasis in fruit flies, which have advantages of genetic tractability, low cost, and high conservation. By screening a collection of genetically modified flies, CD36-like receptors were identified as factors involved in the *Leishmania*-phagocyte interaction. Further testing in CD36-deficient mouse macrophages showed that they did not support parasite proliferation due to the inability of parasites to enlarge the parasitophorous vacuole, a strategy used to avoid toxicity of reactive nitrogen species. The participation of CD36 in the control of the parasitophorous vacuole, which is an altered phagosome, has further implications for diseases such as Alzheimer’s disease, atherosclerosis, and certain bacterial infections where CD36 is a known phagocytic receptor.

## Introduction

Leishmaniasis affects 12 million people in over 98 tropical and subtropical countries or territories [1, 2]. The disease is caused by protozoan parasites of the genus *Leishmania*, which are transmitted by sandflies. In humans, an infection starts with the interaction between promastigote forms of the parasite, delivered by a sandfly bite, and host phagocytes. In the case of *L*. *major* infection, neutrophils in particular are recruited to the bite site and serve as the first host for these parasites, where they differentiate into amastigote forms (3). However, this relationship is temporary because the infection induces apoptotic death of these neutrophils, which stimulates their phagocytosis by dendritic cells and macrophages. The delivery of amastigotes from apoptotic neutrophils to macrophages and dendritic cells as well as the capacity of neutrophils to reduce the recruitment of dendritic cells to the sandfly bite site have been shown to be an important step for the establishment of *L*. *major* infection in the vertebrate host [3–5]. In all types of leishmaniasis, infection is perpetuated in macrophages and the symptoms associated with leishmaniasis are promoted through sequential cycles of intracellular amastigote replication, lysis, and infection of naïve cells.

Host cell receptors engaged during *Leishmania* infection modulate the innate and acquired immune responses. Multiple macrophage receptors are implicated in *Leishmania* recognition, making the study of individual components challenging. The Fcγ receptor was the first of these receptors to be studied in depth; Fcγ receptors bind to antibody-opsonized amastigotes to promote their internalization. This interaction favors infection by inducing the secretion of the anti-inflammatory cytokine IL-10 [6–10]. Similar to Fcγ receptors, complement receptor 3 increases the efficiency of infection and reduces the cellular response against promastigote forms [11, 12]. Parasite recognition by the phosphatidylserine receptor and DC-SIGN are also implicated in down modulating cellular responses while fibronectin and mannose-fucose receptors are examples of receptors that bind to parasites and favor phagocytosis [13–17]. On the other hand, it has been shown that Toll-like receptors (TLR) are required to induce a protective cellular response observed in resistant mouse strains [18–21]. However, the inventory of host factors that are engaged during *Leishmania* infection is still incomplete, limiting our knowledge about host cell signaling pathways essential for leishmaniasis.

Following receptor mediated phagocytosis, the *Leishmania*-containing phagosome rapidly matures into a phagolysosome-like organelle known as a parasitophorous vacuole (PV). The PV size varies among *Leishmania* species: very large PVs that can harbor multiple amastigotes are typical with *Leishmania mexicana* complex (*L*. *amazonensis*, *L*. *mexicana*, *L*. *pifanoi*, *L*. *venezuelensis*) infections. On the other hand, small and tight fitting PVs, which accommodate single amastigotes and split when the parasite replicate, are observed with other *Leishmania* species, such as *L*. *major*. Yet the mechanisms involved in the maturation and maintenance of PVs are largely unknown.

To date, studies have shown that the coordinated fusion of vesicles from both endocytic and secretory pathways, similar to classical phagolysosome maturation, is required for formation of these large PVs. In particular, the PV gradually acidifies and acquires markers of early endosomes (Rab5), late endosomes and lysosomes (Rab7a, MHC class II, LAMP1, LAMP2, M6PR and hydrolases), as well as endoplasmic reticulum markers such as calnexin [22–28]. However, unlike the degradative pathway of classical phagosomes, where the content is degraded and the organelle is recycled, *L*. *amazonensis* PVs quickly enlarge and maintain this enlarged size throughout the life of the infected cell [29]. PV enlargement has been shown to be associated with the high fusogenicity with secondary lysosomes and, to a lesser extent, with the endoplasmic reticulum, however the relative contribution of these organelles for PV formation and the mechanisms that control their fusion to PVs remain unclear [28, 30, 31].

The customization of the PV by *Leishmania* is essential for the acquisition of nutrients and to avoid immune responses. In a series of experiments studying the role of the protein LYST/Beige, Wilson *et al*. (2008) demonstrated that large PVs are essential for *L*. *amazonensis* replication. Cells from mice mutant in *LYST/Beige* have oversized *L*. *amazonensis* PVs with higher parasite replication, while overexpression of *LYST/Beige* inhibited the expansion of PVs and blocked parasite proliferation. Furthermore, large PVs were shown to dilute the intravacuolar concentration of nitric oxide, lowering it to concentrations tolerated by the parasite. The association of PV size with parasite survival has also been related to the fusion of PVs with the endoplasmic reticulum, where interference with SNARE protein functions caused a discrete but significant decrease in PV size and parasite proliferation [28]. These studies highlight the importance of proper enlargement of the PV for *L*. *amazonensis* proliferation.

Here we developed an experimental *Drosophila* model of *Leishmania* infection and used a forward genetic screen to identify novel factors that modulate parasite infection. With this approach, 6 scavenger receptors required for resistance to infection were identified, three of which shared homology with mammalian CD36. We further analyzed the role of CD36 in mammalian models of *L*. *amazonensis* infection and found that CD36 is concentrated in the PV membrane juxtaposed to the posterior end of amastigotes. Moreover, the PV in *CD36* ^-/-^ mouse macrophages was reduced in size, as a consequence of reduced fusion of late endosome and/or lysosomes with the PV, and did not support amastigote replication. Collectively, these studies identify an essential role for CD36 in the maturation of the PV and *Leishmania* survival, and demonstrate that alternative models of infection, such as *Drosophila*, are advantageous for discovery of new host factors involved in the *Leishmania*-host interaction.

## Results

### *Drosophila* infection model

**Fig 1:**
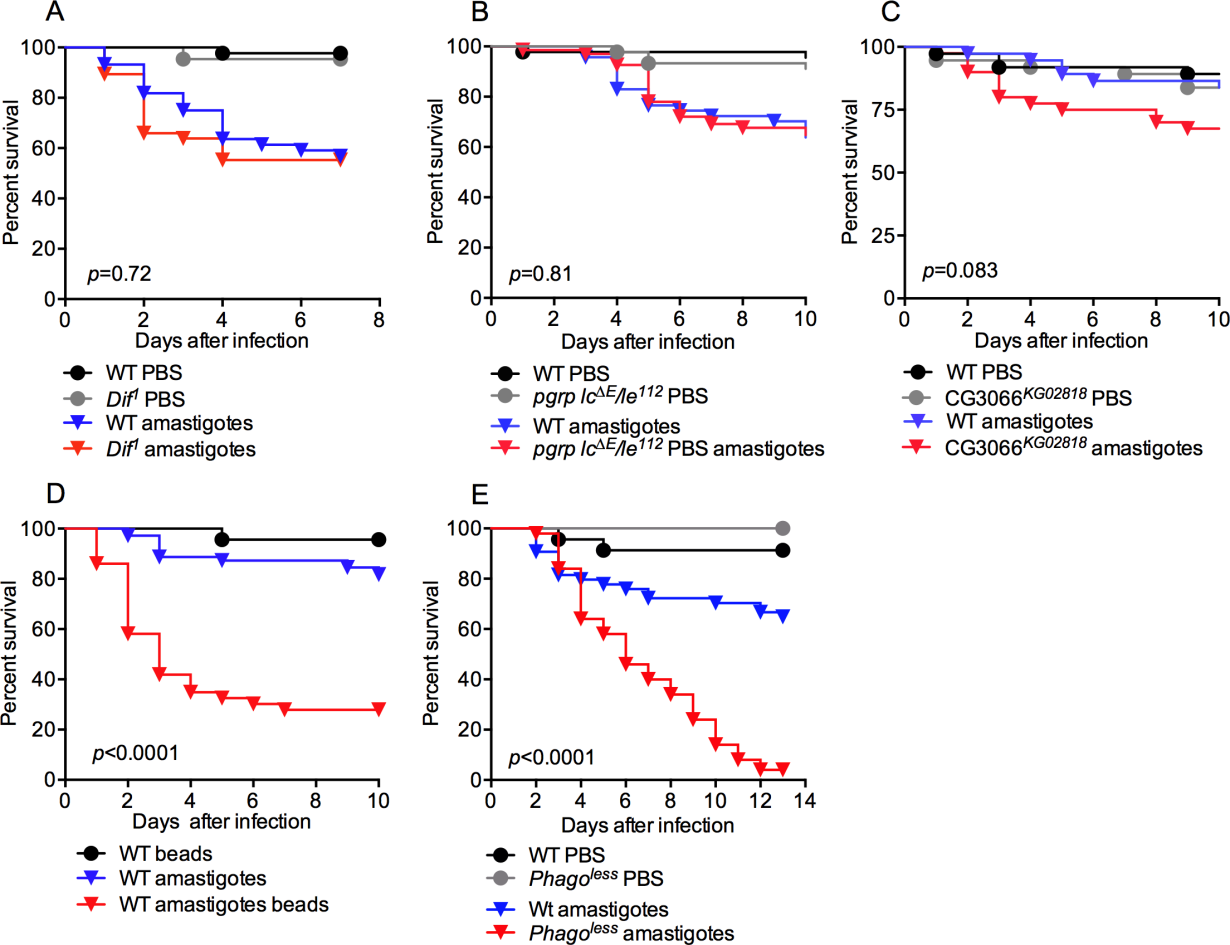
Phagocytosis is required to control *L*. *amazonensis* infection in adult flies. Flies were infected with 40,000 amastigotes by microinjection and survival was monitored daily. Mutant flies in the two main humoral immune pathways, (A) Toll and (B) Imd, or (C) the melanization pathway were not susceptible to infection. (D) The blockage of phagocytosis by injection of polystyrene beads in WT flies or (E) eliminating hemocytes using *phago* ^*less*^ flies (*HmlΔ*Gal4-eGFP, UAS-*Bax*), greatly increased the susceptibility to *Leishmania* infection. At least 60 flies per sample were used in each experiment, graphs are representative of 3 independent assays. The survival curves were analyzed by Log-rank (Mantel-Cox test). *p*-values represent the comparison between WT and mutant (or bead-treated) amastigote-infected flies.

Given the important role of amastigotes in perpetuating *Leishmania* infection and promoting disease, we focused on analyzing the innate interactions between this parasite form and the immune system of the host. As *Drosophila* has proven to be an excellent model system to study such interactions [32], we developed a *Drosophila* model of *L*. *amazonensis* infection, in which opsonin-free amastigotes were isolated from mouse bone marrow-derived macrophage (BMDM) cultures and used to infect adult flies by microinjection. This infection caused only mild lethality, with the death of approximately ~25% of flies between days 3 and 6 post-infection (Fig 1, compare black and blue curves).

The robust survival of ~75% of the *Leishmania*-infected flies suggests the presence of defense mechanisms in *Drosophila* to control these parasites. To identify key defense pathways for host protection, we next infected mutant *Drosophila* that were deficient in known immune defense mechanisms, including humoral immune responses, melanization, or phagocytosis.

### Innate humoral responses and melanization do not affect *Leishmania* infection

The *Drosophila* humoral immune response plays a crucial role in the defense against bacterial and fungal infections through the production of a diverse set of antimicrobial peptides and other factors circulating in the hemolymph. Two NF-κB dependent innate immune pathways control this response, the Toll and Imd (immune deficiency) pathways. We infected flies mutant for either *Dif* or the receptors *PGRP*-*LC*/*LE*, which are essential components of the Toll or Imd pathways, respectively. These mutants had similar survival rates compared to WT flies (Fig 1A and 1B) suggesting that humoral immune signaling, and its ensuing antimicrobial peptide response, does not participate in the defense against *Leishmania* parasites.

Melanization is classically described as a defense mechanism used by arthropods to encapsulate and kill some pathogens such as eggs of parasitoid wasps and the ookinetes of the malarial parasite *Plasmodium berghei* [33, 34]. Melanization is also linked to the effective response against certain bacterial infections [35, 36]. We examined the potential role of the melanization cascade in controlling *Leishmania* infection. Flies deficient in serine protease 7 (CG3066), which are unable to cleave and activate prophenoloxidase and fail to trigger the melanization cascade [37], displayed normal resistance against *Leishmania* infection indicating that this pathway is also not critical for defense against *Leishmania* (Fig 1C).

### Phagocytosis-deficient flies are susceptible to infection

In flies, approximately 95% of hemocytes are plasmatocytes, professional phagocytes responsible for detecting and clearing invading microbes as well as unwanted cells [38]. To evaluate whether plasmatocytes participate in *Leishmania* defense, we ablated the phagocytic ability of plasmatocytes by injecting polystyrene beads into the hemocoel [39]. Polystyrene beads were co-injected with *L*. *amazonensis* amastigotes and fly viability was observed for ten days (Fig 1D). Bead-injected flies were significantly (*p*<0.0001) more susceptible to *Leishmania* infection, with 72% lethality over the course of the 10-day study. This was confirmed using a second approach in which all hemocytes were genetically ablated with the targeted expression of the proapototic protein Bax in plasmatocytes and other blood cells, generating the “*phago* ^*less*^” strain [40]. Similar to the bead-injected animals, this fly strain also showed dramatically reduced survival (*p*<0.0001) following amastigote infection (Fig 1E).

### Plasmatocytes phagocytose *Leishmania* parasites

**Fig 2:**
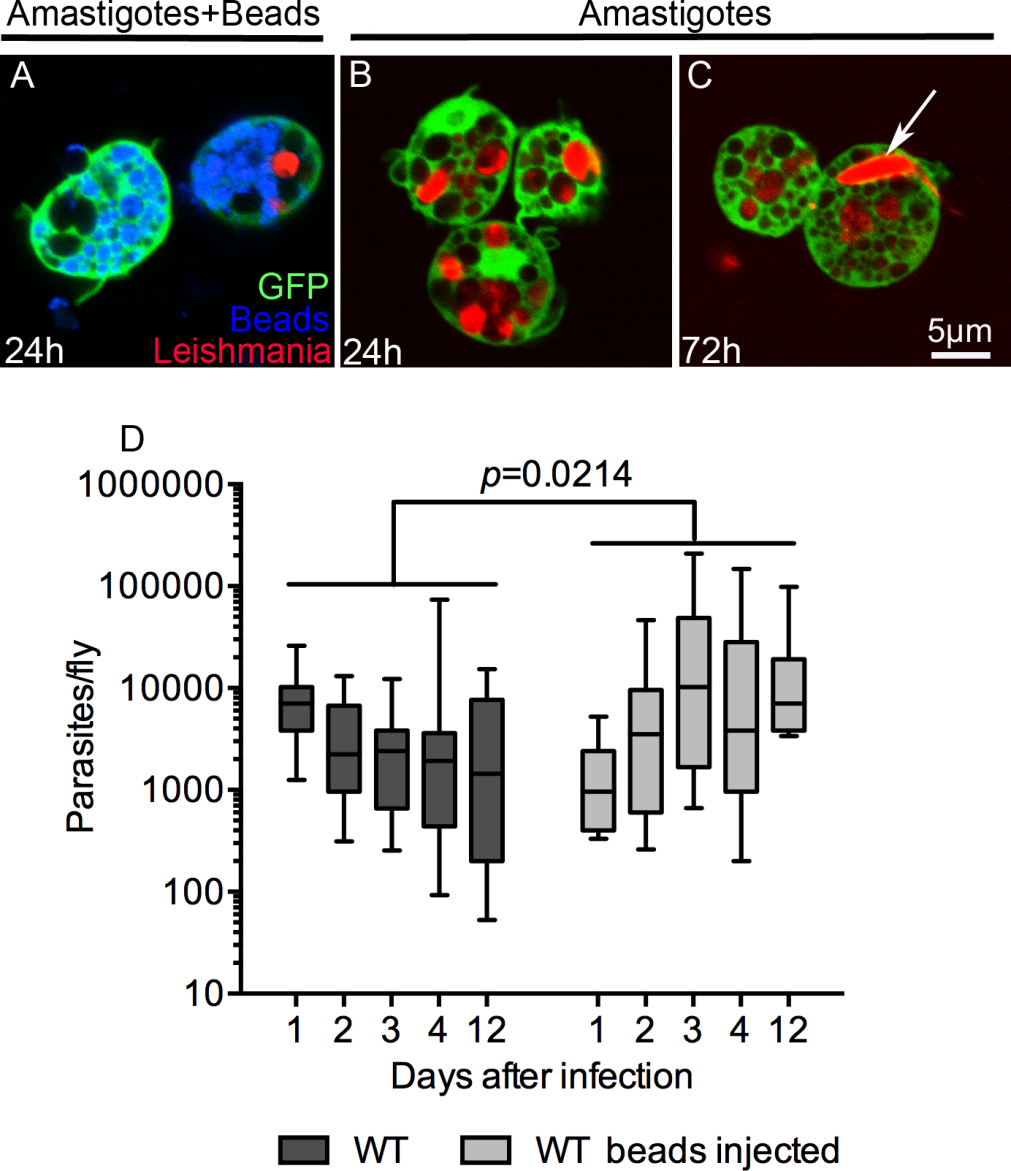
Plasmatocytes from adult flies phagocytose and control *Leishmania* parasites. DsRed-expressing amastigotes were injected alone or with polystyrene beads in *Hml(Δ)*Gal4-eGFP flies. At indicated times, hemocytes were harvested from the hemolymph and analyzed by confocal microscopy. (A) In eGFP-expressing hemocytes from flies injected with polystyrene beads and amastigotes, the cells contained large amounts of intracellular beads (blue) and just one intracellular parasite (red) confirming the inhibitory effect of beads in phagocytosis. (B) 24 h post-injection, plasmatocytes from flies injected only with parasites contained several amastigote forms, while at 72 h after infection some amastigotes differentiated to the long and flagellated promastigote form and were observed inside plasmatocytes (arrow in (C)). (D) Phagocytosis blockage by injection of polystyrene beads increased the parasite burden of flies over time. The control group, flies not injected with beads, prevented parasite growth without completely clearing infection. Parasite burdens were assayed by limiting dilution of single flies, with a minimum of 12 animals per time point. Whiskers represent 10%-90% limits of the sample. Statistical significance was determined by two-way ANOVA.

These results suggest that plasmatocytes and phagocytosis play a critical role in the control of *L*. *amazonensis* infection in *Drosophila*. We hypothesized that plasmatocytes control infection by killing parasites following their phagocytosis. The phagocytosis of parasites was confirmed by microscopic studies of GFP-expressing hemocytes from flies infected with dsRed-expressing *L*. *amazonensis* amastigotes. As expected, flies co-injected with polystyrene beads and amastigotes had plasmatocytes saturated with polystyrene beads but few intracellular parasites at 24 h (Fig 2A); at this same time point, flies injected with amastigotes but not beads had plasmatocytes loaded with numerous parasites (Fig 2B). After 72 h of infection, plasmatocytes contained numerous round amastigotes as well as occasional elongated promastigote forms (Fig 2C). In mammalian macrophages, *L*. *amazonensis* amastigotes proliferate within enlarged PVs, with multiple parasites per vacuole as they replicate. On the other hand, amastigotes in *Drosophila* plasmatocytes remain enclosed in tight fitting phagosomes which rarely contain more than one parasite (Fig 2B and 2C). This suggests that amastigotes cannot effectively manipulate the *Drosophila* phagocytic machinery to create their preferred niche, and instead these hemocytes control the infection through phagocytosis and parasite killing.

### Parasites survive and replicate in susceptible adult flies

We next evaluated whether the high mortality of flies correlated with increased parasite proliferation. The amount of parasites surviving within the host was estimated by limiting dilution of individual flies. Bead-treated flies contained significantly more parasites than untreated flies (Fig 2D). Additionally, while the control flies showed a trend of reduced parasite load over the course of 12 days of infection, the flies defective in phagocytosis had a parasite load that increased over three days post-infection and remained elevated until day 12 (Fig 2D). These data are consistent with the high death rate observed between the 3^rd^ and 6^th^ day of infection of phagocytosis-deficient flies (Fig 1D and 1E). In summary, these data demonstrate that phagocytic activity, and plasmatocytes in general, are critical for controlling parasite proliferation and protecting the fly from death.

### *In vivo* screening identifies CD36 scavenger receptors as a critical component for *Leishmania* defense

**Fig 3:**
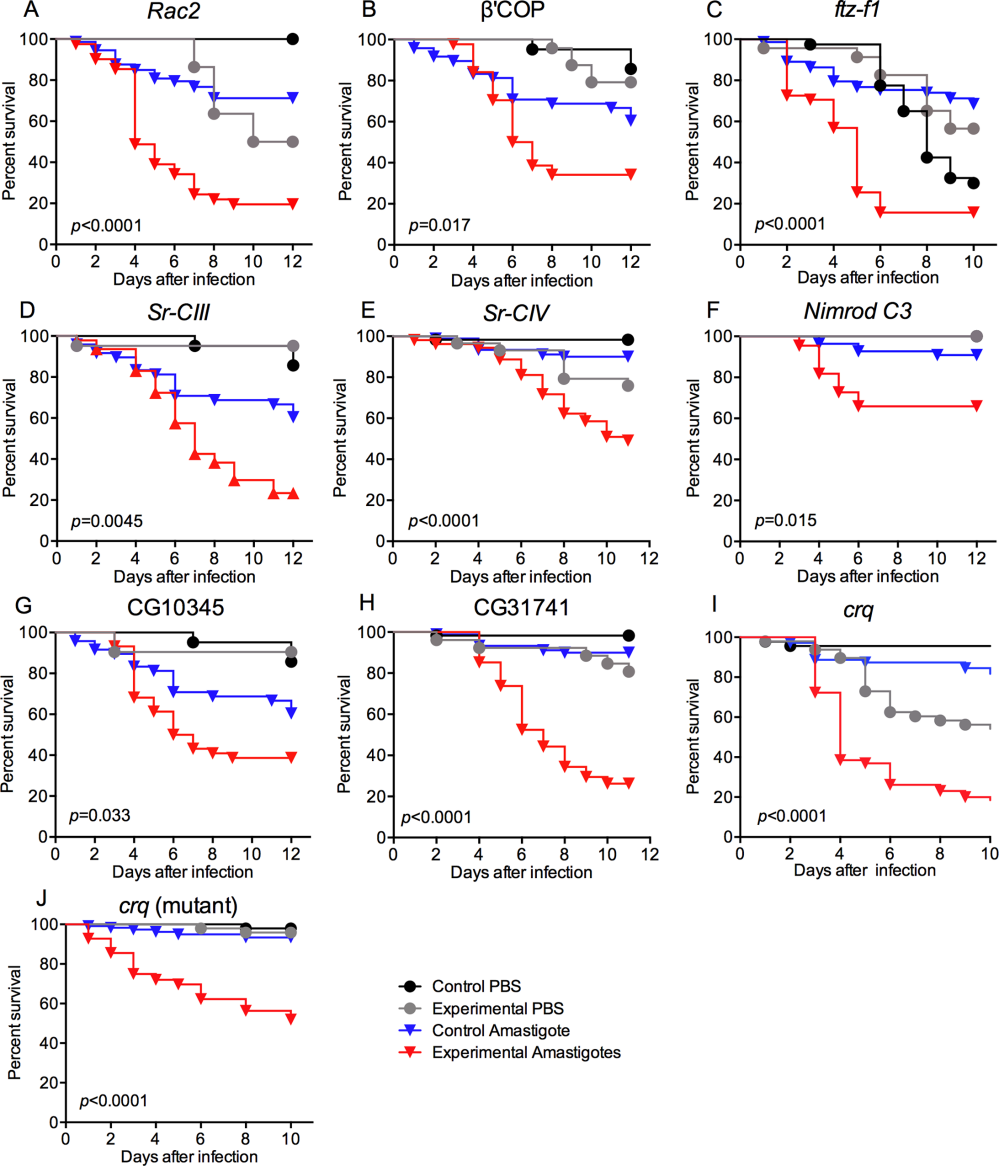
An *in vivo* RNAi screen identified factors required for *Drosophila* resistance to *L*. *amazonensis* infection. Nine hemocyte-expressed factors were identified as being required to control *Leishmania* infection after screening a collection of 32 RNAi or mutant lines (Table 1). Three general phagocytic factors (Rac2, β’COP and ftz-f1, A-C), and six scavenger receptors (D-I) were identified. The receptor hits fell into three classes: two were Class C receptors (a group unique to some Diptera) (D, E), one Nimrod type (F), and three CD36-like receptors (SR-B family): CG10345, CG31741 and croquemort (G-J). Control flies for all RNAi assays included the *Hml(Δ)*-Gal4 driver but no UAS-RNAi (A-I), while heterozygous flies were used as controls for the *crq* mutant (J). At least 60 flies were used per sample, survival curve analysis was performed using a Log-rank (Mantel-Cox) test, indicated *p*-values were determined from the comparison of control and RNAi/mutant-infected flies.

Given the importance of phagocytosis in controlling *Leishmania* infection, the *Drosophila* model was then used to screen for hemocytes genes required for anti-parasite defense. We analyzed a collection of flies carrying RNAi constructs targeting a curated set of potential phagocytic genes. Targeted factors included the core phagocytic machinery and hemocyte-specifying factors, such as Rac2 or FTZ-F1, as well as a collection of 25 receptors linked to phagocytosis, including members of the Scavenger Receptors Class B (*i*.*e*., CD36-like), Class C (an insect-specific group) and Class F (rich in EGF repeats such as Nimrods, Eater, Draper and Slow down (Table 1)). The knockdown of these genes was targeted specifically to hemocytes using the *Hml(Δ)*-Gal4 driver. Consistent with the role of phagocytosis in the *Drosophila* defense against *Leishmania* infection, this approach identified several essential phagocytic components, including Rac2, Coat Protein (coatomer) ß’ and the ftz transcription factor 1 (Fig 3A–3C, Table 1). In addition, multiple scavenger receptors were found to be important for defense against *L*. *amazonensis* infection: SR-CIII and SR-CIV, Nimrod C3, and three CD36-like receptors (or SR-Bs), namely CG10345, CG31741, and Croquemort (Fig 3D–3J and Table 1).

**Table 1:** *Drosophila in vivo* screening for phagocyte factors protective against *L*. *amazonensis* infection. Gene knockdown was targeted to hemocytes by crossing a fly line carrying the *Hml*(Δ)-Gal4-driver to UAS-RNAi-expressing flies. Croquemort and draper mutant lines were also analyzed, as indicated. The 3^rd^ column presents the percentage of assays where the survival of the experimentally-infected flies was significantly reduced compared to PBS-injected RNAi flies and to control amastigote-infected flies (*p*<0.005), the total number of independent survival assays are presented in parentheses. Fly lines that were susceptible in more than 60% of the assays were considered hits (bold). The hyper-susceptibility of fly lines deficient in Rac2, ftz-f1 and ß’COP, demonstrates that the approach identifies known phagocytic factors. The comparison of the curves was performed using a Log-rank (Mantel-Cox) test. Representative survival curves of susceptible flies are presented in Fig 3.

Gene name or symbol	Protein family or feature	% assays with reduced survival (n)
**croquemort**	**Scavenger receptor Class B**	**71 (7)**
**croquemort (mutant)**	**Scavenger receptor Class B**	**75 (4)**
**CG31741**	**Scavenger receptor Class B**	**62 (8)**
**CG10345**	**Scavenger receptor Class B**	**71 (7)**
debris buster	Scavenger receptor Class B	25 (4)
CG2736	Scavenger receptor Class B	33 (3)
epithelial membrane protein	Scavenger receptor Class B	33 (3)
peste	Scavenger receptor Class B	0 (2)
CG7227	Scavenger receptor Class B	0 (7)
santa-maria	Scavenger receptor Class B	0 (2)
Sensory neuron membrane protein 1	Scavenger receptor Class B	0 (3)
CG3829	Scavenger receptor Class B	0 (2)
**Scavenger receptor class C, type III**	**Scavenger receptor Class C**	**75 (8)**
**Scavenger receptor class C, type IV**	**Scavenger receptor Class C**	**67 (6)**
Scavenger receptor class C, type II	Scavenger receptor Class C	25 (8)
Nimrod A	Scavenger receptor Class F-like	33 (3)
Nimrod B1	Scavenger receptor Class F-like	0 (3)
Nimrod B2	Scavenger receptor Class F-like	0 (2)
Nimrod B5	Scavenger receptor Class F-like	0 (2)
Nimrod C1	Scavenger receptor Class F-like	33 (3)
Nimrod C2	Scavenger receptor Class F-like	33 (3)
**Nimrod C3**	**Scavenger receptor Class F-like**	**63 (8)**
Nimrod C4	Scavenger receptor Class F-like	33 (3)
eater	Scavenger receptor Class F-like	0 (3)
slowdown	Scavenger receptor Class F-like	0 (2)
draper (mutant)	Scavenger receptor Class F-like	0 (2)
**ftz transcription factor 1**	**Nuclear hormone receptor**	**100 (3)**
**Rac2**	**Ras GTPase**	**100 (3)**
**Coat Protein (coatomer) β'**	**WD repeats**	**75 (4)**
phagocyte signaling impaired	Tetratricopeptide domains repeat	33 (3)
SCAR	Actin-binding protein	0 (2)
Rab5	Ras GTPase	0 (2)

### Knockdown of CD36-like receptors reduces phagocytosis and increases parasite burden in flies

**Fig 4:**
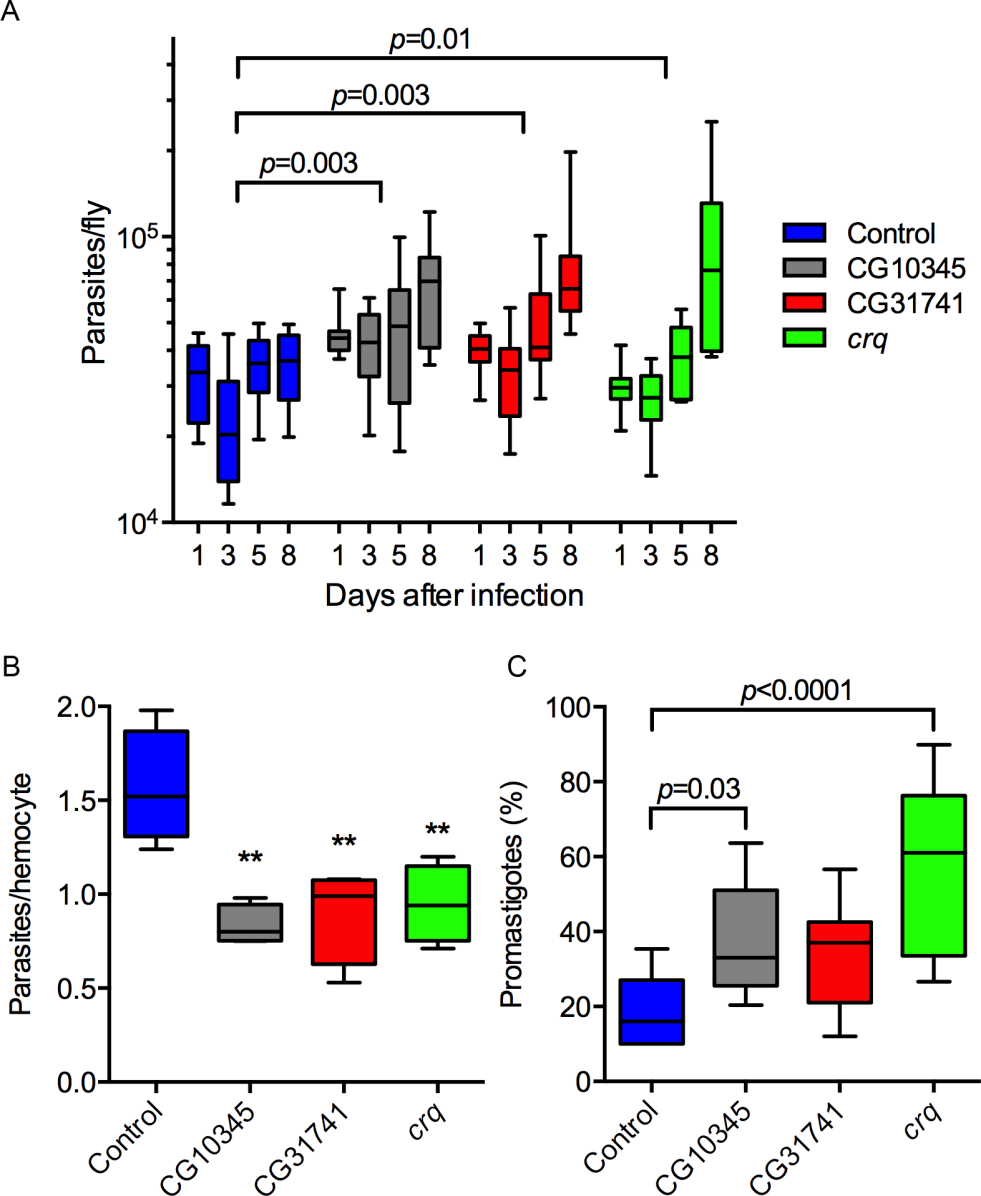
Knockdown of CD36-like receptors in hemocytes reduced phagocytosis and favored *Leishmania* proliferation in flies. (A) While control flies did not allow parasite proliferation, the RNAi lines had a significant increase in parasite burden, determined by two-way ANOVA, n = 10. (B) Hemocytes were harvested from adult flies 1 h post-infection for microscopic scoring of infection. The knockdown of CD36-like receptors reduced the number of intracellular parasites (**: *p* < 0.01 one-way ANOVA, n = 4). (C) Two out of three RNAi fly lines had a higher percentage of promastigotes at 8 days post-infection. One-way ANOVA, n = 10. Whiskers represent 10%-90% limits of the sample. Representative data from two independent assays.

Because CD36-deficient flies were more susceptible to *Leishmania* infection (Fig 3), we next investigated if this susceptibility was linked to an increase in parasite burden and defects in phagocytosis. While the parasite load of control flies did not increase during infection, a sharp increase in parasites was observed in flies expressing RNAi for CD36-like receptors (Fig 4). Next, to investigate the role of CD36 scavenger receptors in phagocytosis, the number of parasites associated with hemocytes was scored 1 h post-infection. Notably, hemocytes from RNA flies for *Crq*, CG31741, and CG10345 contained significantly less intracellular parasites, compared to WT hemocytes (Fig 4B). These data support our hypothesis that the expression of each of these three CD36-like scavenger receptors in *Drosophila* hemocytes is critical for control of *Leishmania* infection by phagocytosis.

Next we evaluated which parasite stage is proliferating in the susceptible flies. At day one of infection all parasites are in the amastigote form, in all genotypes. However, in control flies by day three 15% of parasites are promastigotes and this increases to 20% by day eight. With knockdown of CG10345 or *crq*, promastigote levels significantly increased to 40% or 60%, respectively (Fig 4C). However, total parasite load did not correlate with the degree of promastigote differentiation/replication.

### Mammalian CD36 relocalizes to the PV membrane juxtaposed to the amastigote contact point

**Fig 5:**
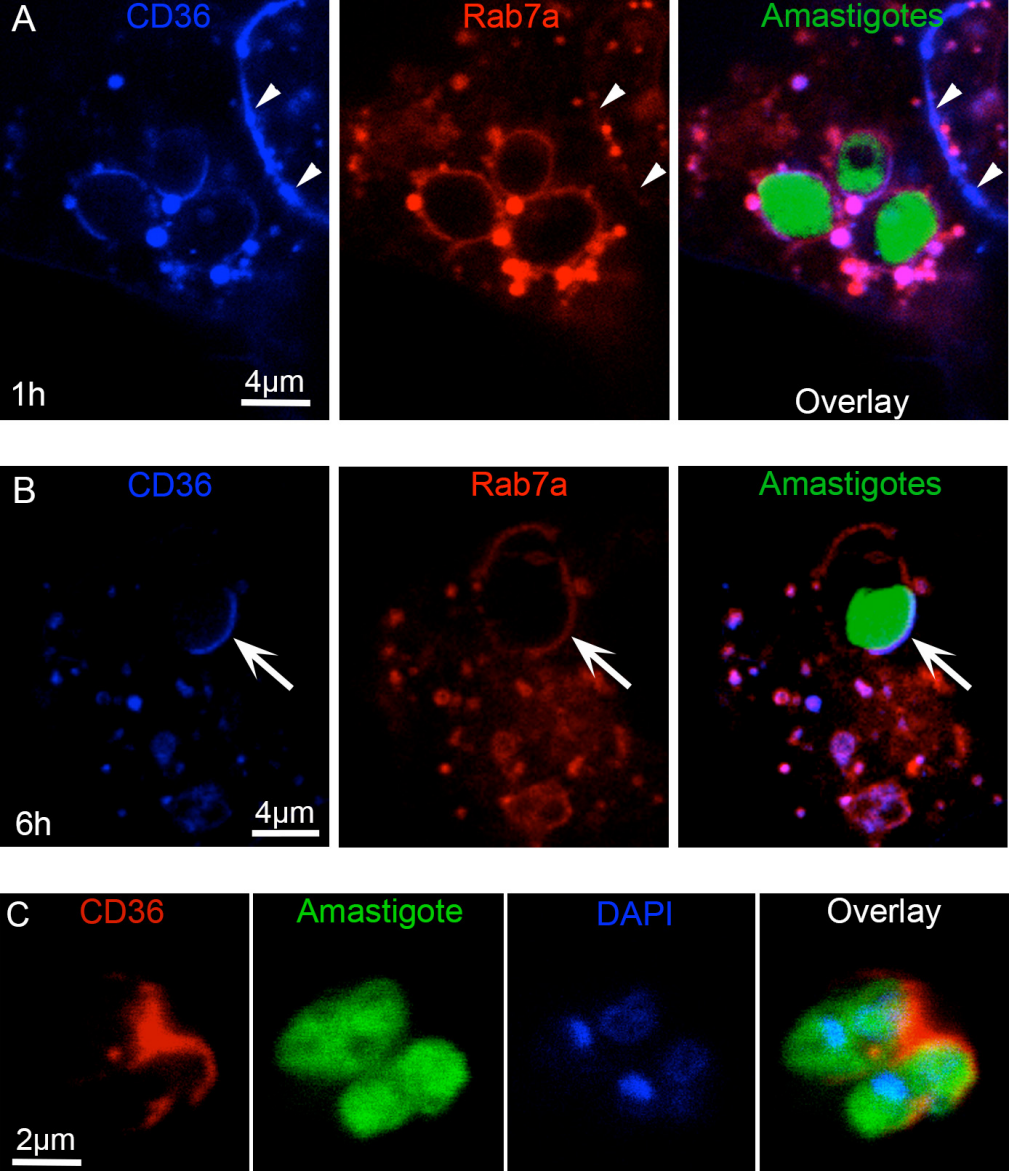
CD36 concentrates at the PV membrane juxtaposed to parasites. 293T cells coexpressing mCherry-Rab7a and mCerulean3-CD36 were infected with GFP-expressing parasites and analyzed by confocal microscopy. (A) In the first hour of infection, CD36 was mainly co-localized with Rab7a in positive cytoplasmic vesicles, many of which were adjacent to the PV, and at cell membranes (arrowheads) and the entire PV. (B) By 6 h post-infection, CD36 was highly concentrated at the PV membrane juxtaposed to the amastigotes (arrows) while Rab7a was evenly distributed. (C) Amastigotes harvested from mCherry-CD36 expressing 293T cells by mechanical disruption retained CD36 (red) at the posterior pole indicating a stable interaction 24 h after infection.

Given the results with knockdown of CD36 homologs in the *Drosophila* model, we investigated the role of this scavenger receptor in the interaction between *L*. *amazonensis* and mammalian cells. The localization of mCerulean3-tagged CD36 during amastigote infection of human 293T cells was analyzed. By one hour post-infection, CD36 was widely distributed on the cell membrane and co-localized with mCherry-Rab7a at numerous cytoplasmic vesicles and at the PV membrane (Fig 5A). Notably, at 6 h post-infection, when the PV is expanding, CD36 concentrated at the PV membrane juxtaposed to the parasite point of contact (Fig 5B). This suggests that localization of CD36 on the PV membrane is regulated in response to the amastigote.

To further demonstrate the close association of CD36-positive PV membranes and parasites, we studied the association of CD36 with amastigotes harvested from mCherry-CD36 expressing 293T cells infected with GFP-expressing parasites. The intracellular parasites were isolated free of host cells 24 h after infection by mechanical disruption and centrifugation. These amastigotes retained a strong mCherry-CD36 signal at their posterior end (Fig 5C), suggesting a persistent CD36-parasite interaction in infected cells.

A time-lapse confocal micrograph of amastigote-infected mCherry-CD36 293T cells (S1 Movie) showed the dynamic fusion of the late endosomes and lysosome in cells infected for 6 h and analyzed after a 15 min pulse of pHrodo-Dextran. When endocytosed, pHrodo serves as a marker of late endosomes and lysosomes because it becomes highly fluorescent in the acidic environment of these organelles. PV regions juxtaposed to parasites and rich in CD36 were associated with numerous acidic (pHrodo positive), mCerulean3-CD36 positive organelles. Although it was not possible to differentiate between vesicles fusing with PV from those simply moving out of the focus plane, the gradual decrease in the number of endocytic vesicles along with the increase of pHrodo signal into the PV is a clear indication of fusion.

### CD36 is required for PV biogenesis

**Fig 6:**
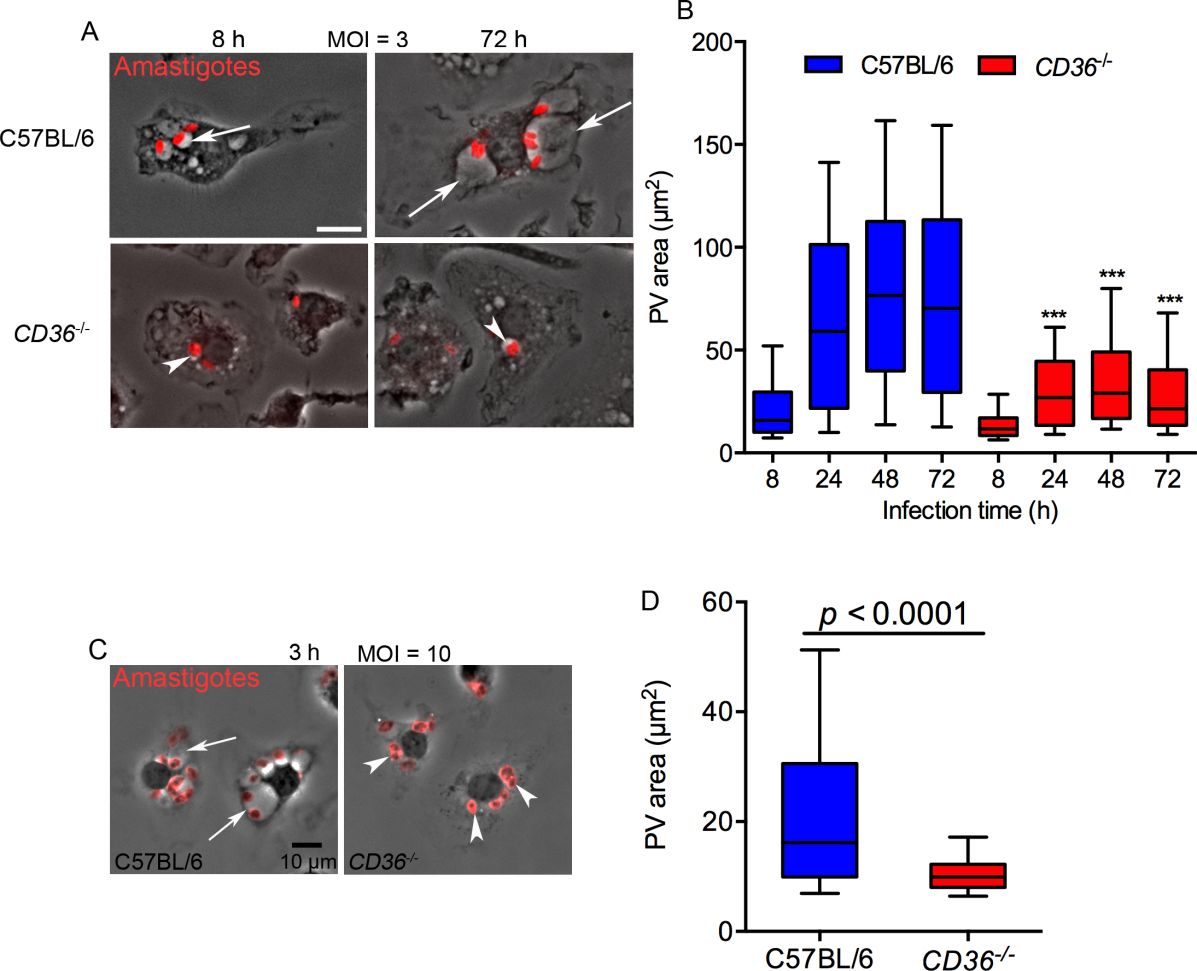
CD36 is required for expansion of PVs. (A) C57BL/6 BMDMs had larger PVs (arrows) than CD36-deficient BMDMs (arrowheads), Bar: 10μm. (B) The area of PVs from *CD36* ^-/-^ BMDMs was significantly smaller than those of C57BL/6 BMDMs between 24 h and 72 h after infection using an MOI = 3. Results from 2 independent experiments, the whiskers in the box plot graph represent 10–90% of PVs (n = 120, ***:*p*<0.001 two-way ANOVA, Tukey’s multiple comparison test). (C-D) Differences in PV size between WT and *CD36* ^*-/*-^ BMDMs are noticeable at 3 h post-infection using an MOI = 10. Unpaired t-test, representative result of 2 independent experiments (n = 200 PVs).

The intriguing recruitment and localization of CD36 on the PV of 293T cells, led us to examine the phenotype of amastigote infected *CD36* ^*-/*-^ mouse bone marrow derived macrophages (BMDMs), a more relevant cell type for *Leishmania* studies. *L*. *amazonensis* parasites, as well as other members of the *L*. *mexicana* subgenus, invade macrophages by receptor-mediated phagocytosis and induce the formation of an enlarged PV in WT cells (Fig 6). We infected BMDMs derived from WT (C57BL/6) or *CD36* ^*-/*-^ mice with a multiplicity of infection (MOI) of three parasites per macrophage and observed that the size of the PVs was similar between *CD36* ^*-/*-^ and WT at 8 h post-infection but by 24 h the PVs of WT macrophages were significantly larger (Fig 6A and 6B). The difference peaked at 48 h when the area of PVs from WT macrophages reached 62 μm^2^ while the area of PVs from *CD36* ^*-/*-^ macrophages was only 16 μm^2^. From 48 h to 72 h the size of PVs did not change significantly (Fig 6B).

At an MOI = 3, PV expansion occurs slowly and this may contribute to the delay in the appearance of clear differences between WT and CD36-deficient BMDMs. Therefore, BMDMs were infected at an MOI = 10, which induces a quicker expansion of the PV. Using this condition, WT cells had enlarged PVs by 3 h post infection, while the PVs in the CD36-deficient macrophages remained small (Fig 6C and 6D), suggesting that CD36 is directly involved in the expansion and maintenance of *L*. *amazonensis* PVs.

### *CD36* ^-/-^ macrophages are infected but do not support *L*. *amazonensis* proliferation

**Fig 7:**
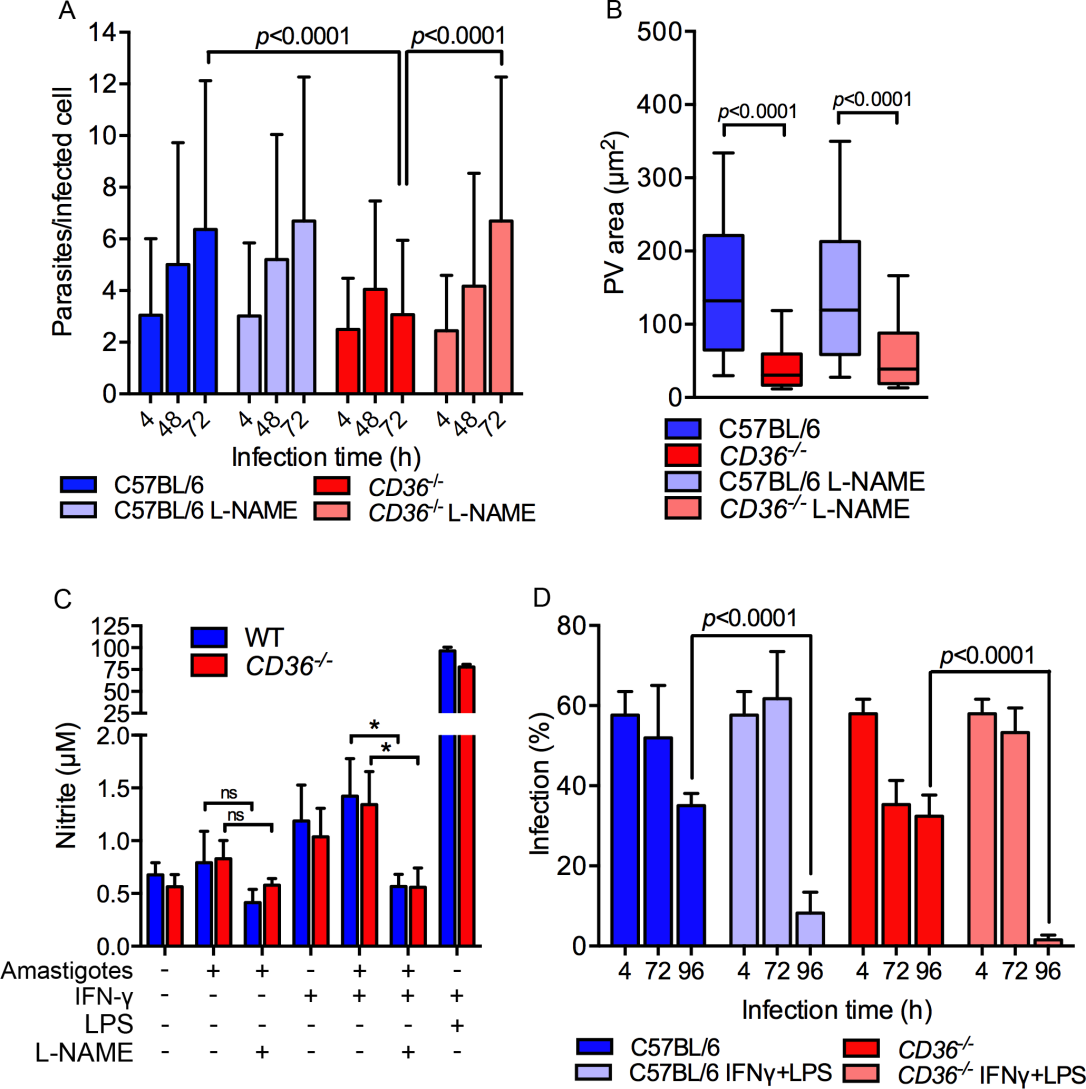
Local nitric oxide (NO) toxicity arrests parasite proliferation in *CD36* ^-/-^ macrophages. (A) Compared to WT cells, *CD36* ^-/-^ macrophages did not support parasite replication, which is clearly observed at 72 h post-infection; however, the treatment with the iNOS inhibitor L-NAME restored parasite replication, n = 100, two-way ANOVA, Tukey’s multiple comparison test, Error bars represent the standard deviations. (B) The parasite proliferation in *CD36* ^*-/*-^ L-NAME-treated cells did not induce PV enlargement. n = 200, t-test analysis. (C) Amastigote-infected macrophages produced limited NO in WT and *CD36* ^-/-^ BMDMs. L-NAME treatment significantly reduced NO production in IFN-γ treated cells (*: *p<*0.05 unpaired t-test). (D) Upon activation with LPS and IFN-γ, *CD36* ^-/-^ and WT BMDMs were capable of killing intracellular parasites. Macrophages were activated with addition of IFN-γ and LPS 24 h post-infection and the percentage of infected cells scored at indicated times. Both C57BL/6 and *CD36* ^-/-^ activated macrophages drastically reduced the infection by 96 h. Representative results of 2 independent experiments each using 3 replicates of 200 cells each. Statistical significance was determined by one-way ANOVA and Tukey’s multiple comparison test, error bars represent the standard deviations.

We next evaluated if parasites were able to establish a replicative niche in the small PV of *CD36* ^-/-^ macrophages. The percentage of infected cells and the average number of parasites per infected cell were similar in WT and *CD36* ^-/-^ macrophages at 4 h post-infection, indicating that CD36 is not essential for parasite phagocytosis (Fig 7). However, while the replication rate of the parasite was similar in *CD36* ^-/-^ and WT macrophages during the first 48 h of infection, by 72 h parasite proliferation in *CD36* ^-/-^ macrophages was significantly reduced compared to WT suggesting a key role for CD36 in amastigote proliferation (Fig 7A).

Wilson *et al*. (2008) reported that the large PV of *L*. *amazonensis*-infected cells dilutes the concentration and toxicity of nitric oxide (NO) inside the PV, thereby allowing parasite replication [41]. To evaluate whether the impaired proliferation of parasites in the small PV of *CD36* ^-/-^ macrophages was related to the NO toxicity, macrophages were treated with N-nitro-L-arginine methyl ester (L-NAME), an inhibitor of NO synthase, prior to amastigote infection. Notably, the inhibitor rescued the number of parasites in *CD36* ^-/-^ macrophages at 72 h post-infection (Fig 7A), confirming the role of NO in restraining parasite proliferation in the small PVs of *CD36* ^-/-^ macrophages. On the other hand, L-NAME had no effect on PV size (Fig 7B) and significantly reduced the NO levels of infected macrophages stimulated with IFN-γ (Fig 7C). However without IFN-γ stimulation, the levels of NO remained low and the effect of L-NAME on this low level of NO was not significant (Fig 7C). Therefore, L-NAME treatment likely protects parasites from the local NO production that is thought to occur at the PV surface [42].

Intriguingly, while local NO impaired parasite replication in the small PV of CD36-deficient macrophages, GFP-expressing parasites were observed for at least 4 days in these cells, suggesting that they could not mount a sterilizing anti-parasitic response. The production of reactive nitrogen species in IFN-γ/LPS-stimulated macrophages is important to the clearance of intracellular parasites [43]. Uninfected *CD36* ^*-/*-^ macrophages produced WT levels of NO following IFN-γ and LPS (Fig 7C). Consistent with this finding, IFN-γ/LPS treatment 24 h post-infection caused a nearly complete clearance of intracellular parasites in both WT and *CD36* ^-/-^ macrophages by 96 h (Fig 7D).

Together, these data confirm that *CD36* ^-/-^ macrophages maintain IFN-γ/LPS-inducible anti-parasitic activity and argue that the large PV of *L*. *amazonensis* protects amastigotes from the local production of NO, but not against the high concentrations generated by fully activated macrophages.

### *CD36* ^-/-^ macrophages present normal incorporation of fatty acids and cholesterol

CD36 is involved in the transport of long chain fatty acids and cholesterol [44, 45]. Therefore, we investigated if *CD36* ^-/-^ macrophages exhibited any defects in the transport of these lipids to the PV or to the parasites that might relate to the formation of PVs. The incorporation of BODIPY FL C12 and CholEsteryl BODIPY was measured by flow cytometry at 2 h of continuous presence of the probes in the culture. The incorporation for both probes was similar in uninfected WT and *CD36* ^-/-^ macrophages, and both probes were also similarly incorporated into *L*. *amazonensis* amastigotes within the PV of WT and *CD36* ^-/-^ macrophages (S1 Fig), indicating that these lipids are trafficking normally in the absence of CD36.

### Undersized PVs are not related to modulation of *Beige* expression or TLR2/4 signaling

In a previous study, *Beige* was shown to regulate the *L*. *amazonensis* PV size [41]. In particular, these authors concluded that the expression of *Beige* is triggered by *Leishmania* infection, as a defense mechanism that reduces the PV size and increases the intravacuolar NO concentration for more effective parasite killing. *Beige* mRNA levels were quantified by qPCR in WT and *CD36*-deficient macrophages after infection with *L*. *amazonensis* amastigotes. If *Beige* was related to the undersized PV in *CD36* ^*-/*-^ macrophages we would expect higher expression of this gene, however we did not detect significant differences compared to WT (S2A Fig).

CD36 has also been implicated as a co-receptor for TLR2/6 and TLR4/6 heterodimers, which are involved in triggering the inflammatory response following stimulation with *Staphylococcus aureus* [46] or oxidized low-density lipoprotein [47, 48]. To determine if the small PVs in CD36-deficient macrophages were caused by an inefficient CD36-TLR signaling axis, the PV size was measured in immortalized macrophages (iMO) from mutant mice lacking the adaptor protein MyD88 or both MyD88 and TRIF. As expected, CD36-deficient iMOs had smaller PVs, but *MyD88* single or *MyD88/TRIF* double knockout iMOs contained PVs with areas comparable to WT iMOs (S2B Fig), demonstrating that the undersized PVs of CD36-deficient macrophages is not related to TLR signaling.

### Lentiviral expression of *CD36* rescues the undersized PV phenotype of *CD36* ^-/-^ macrophages

**Fig 8:**
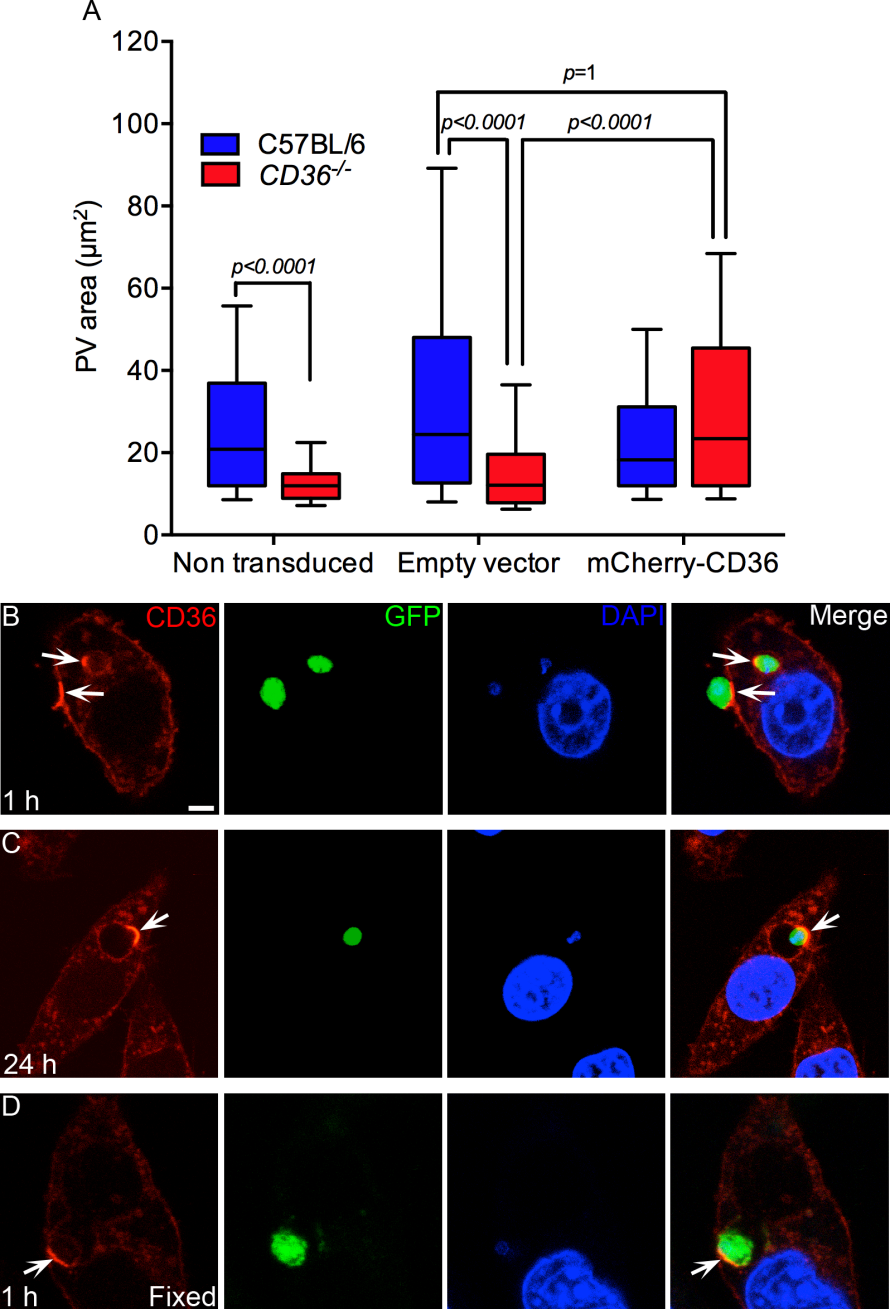
Lentiviral expression of mCherry-*CD36* in *CD36* ^-/-^ iMOs rescues the small PV phenotype and shows the recruitment of CD36 to PVs. (A) *CD36*-deficient iMOs exhibited smaller PVs than WT cells at 48 h of infection. The expression of mCherry-CD36 via a transducer lentiviral expression vector, restored the WT PV size to the *CD36* ^-/-^ iMOs. A representative result of 2 independent experiments is shown, n = 200. Statistical significance was determined by Dunn's multiple comparison test. (B-D) *L*. *amazonensis* amastigotes induced clustering of CD36 at the cell membrane and in the PV. (B) Clusters of CD36 present in the cell membrane at the region of contact with an extracellular amastigote and in the PV of another intracellular parasite (arrows). (C) By 24 h after infection, the clusters of CD36 are always found in the PV membrane where the parasite is anchored by its posterior pole (arrow). (D) Formaldehyde fixed amastigotes also induce the clustering of CD36 in the PV (arrow). Bar: 5 μm. (C-D) At least 25 parasites were analyzed in 2 independent assays.

To test whether overexpression of CD36 could rescue the undersized PV phenotype of *CD36* ^-/-^ macrophages infected with *L*. *amazonensis*, *CD36*-deficient iMOs were engineered to express mCherry-tagged CD36 and infected with *L*. *amazonensis* amastigotes. As shown in Fig 8A, *CD36* ^-/-^ iMOs exhibited small PVs, consistent with our observations in primary macrophages. The expression of mCherry-CD36, via a stable transduction of a lentiviral expression vector, restored the PV size of amastigote-infected *CD36* ^-/-^ iMO to WT levels.

### Amastigote contact with the plasma membrane induces CD36 aggregation

While infection of 293T cells induced the aggregation of CD36 in the PV within a few hours of infection, mCherry-CD36 expressing iMOs exhibited much more rapid CD36 clustering. Immediately following amastigote contact with the iMO, CD36 was observed clustering on the plasma membrane at the amastigote contact site, as part of the phagocytic cup (Fig 8B). These CD36 clusters were then incorporated, along with some of the plasma membrane, into the developing PV during amastigote entry (Fig 8B). During the first 6 h of infection, the CD36 clusters were not associated with a specific pole of the parasite, but were always localized at the point of contact. Later, with the enlargement of the PV, the posterior pole of most parasites aligns to contact the PV membrane, reorienting the CD36 clusters to this region of the parasite (Fig 8C). The attachment and polarization of amastigotes inside PV is typical for *L*. *amazonensis* [23, 26, 29].

We next asked whether the amastigote actively induces the recruitment of CD36 to the cell membrane surface by secretion of virulence factors or by passive receptor-ligand interaction. Formaldehyde-fixed amastigotes also induced CD36 clustering, demonstrating that molecules on the surface of the parasite are responsible for CD36 recruitment (Fig 8D). However, the formaldehyde-fixed amastigotes were degraded by macrophages within 6 h of infection and the CD36 aggregates were dispersed.

### Promastigotes of *L*. *amazonensis* do not induce CD36 clustering

**Fig 9:**
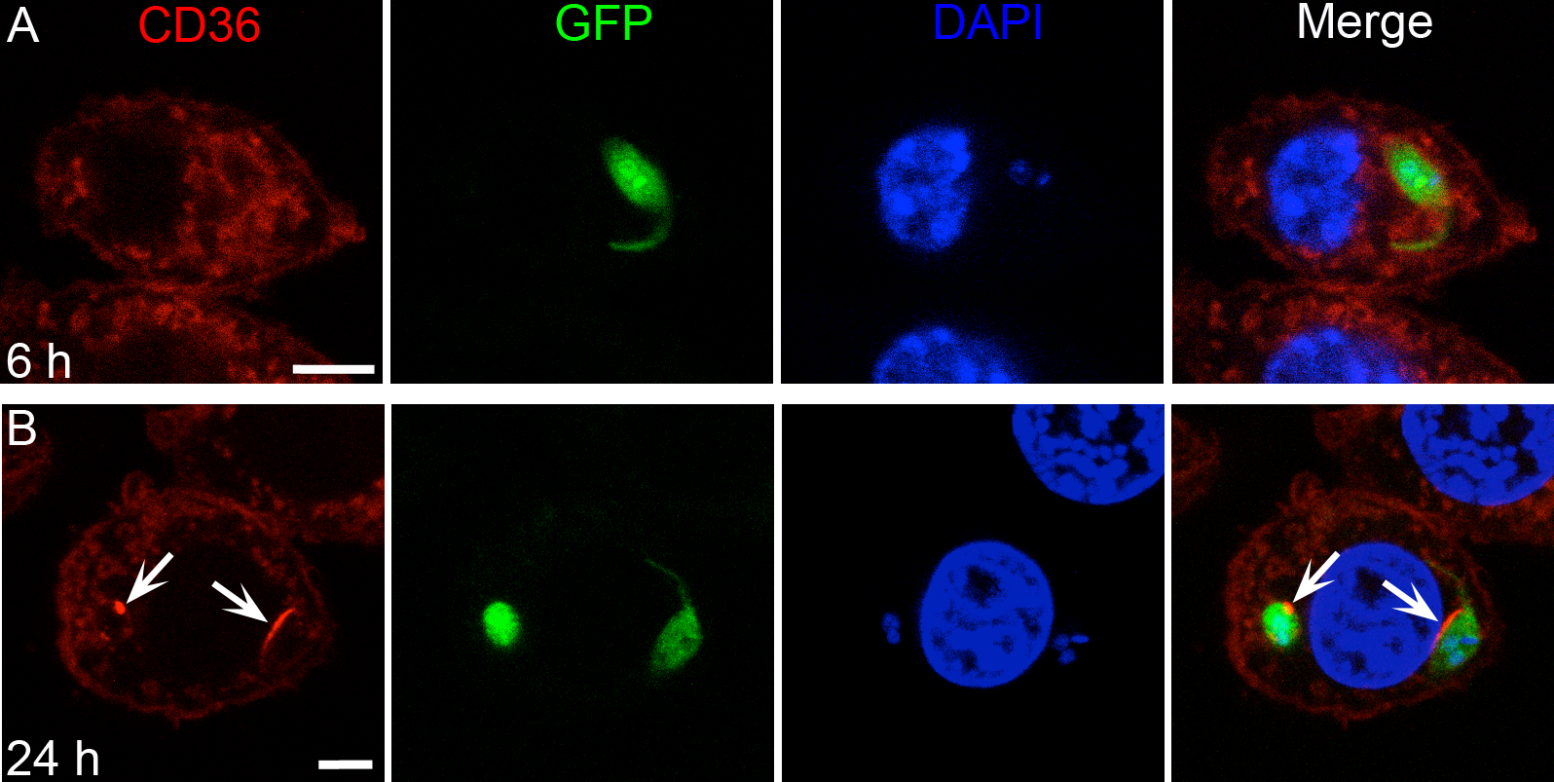
In macrophages, CD36 recruitment is delayed in PVs containing *L*. *amazonensis* promastigotes. (A) PV-containing promastigotes did not exhibit clustering of CD36 up to around 6 h after infection, however, by 24 h (B) some PVs presented accumulation of CD36 (arrows), probably associated with the differentiation to amastigote forms. Bar: 5 μm.

Promastigotes are the initial infective form of the parasite and are also internalized by macrophages. To test whether promastigotes induce CD36 recruitment, we infected iMOs expressing mCherry-CD36. Notably, unlike amastigotes, promastigote cell contact and internalization did not recruit CD36 within the first 6 h of infection (Fig 9A). However, starting at 6 h, PV containing promastigotes (or differentiating amastigotes) gradually acquired CD36, which increased up to 24 h (Fig 9). Note, the promastigote PVs were still small and tight fitting, indicating a delay in PV enlargement compared to infections initiated with amastigotes.

### *L*. *major* amastigotes do not recruit CD36 and proliferate normally in *CD36* ^-/-^ macrophages

To further investigate the role of CD36 clustering in *Leishmania* infection, we studied CD36 dynamics in response to *L*. *major*, which proliferate in small single parasite PVs. Amastigotes were harvested from mouse footpad lesions and used immediately to infect iMOs expressing mCherry-CD36. Confocal microscopy demonstrated that CD36 was not enriched in the small PVs of *L*. *major* at either 6 h or 24 h post-infection (S4A and S4B Fig).

The absence of CD36 clustering in *L*. *major* tight fitting PVs suggests that the receptor is not involved in this infection. However, to further evaluate the participation of CD36 in *L*. *major* proliferation, the parasite loads of WT and *CD36* ^*-/*-^ macrophages were monitored during a time course of infection. The number of parasites recovered in WT and *CD36* ^*-/*-^ macrophage cultures was similar throughout the infection (S4C and S4D Fig). The initial parasite load at 3 h of infection was relatively high because both infective and non-infective parasites were recovered. The following 3 days, both WT and *CD36* ^*-/*-^ macrophages had low parasite levels because only a small proportion of infective promastigotes survived. Parasite proliferation was detected by 96 h post-infection in both genotypes, indicating that lack of CD36 does not impair *L*. *major* proliferation.

### PV enlargement is not related to a phosphatidylserine-CD36 interaction

CD36 has been reported to induce cell membrane fusion through binding to phosphatidylserine [49]. In this model, the interaction of CD36 from one cell with phosphatidylserine on the surface of another, promotes the fusion of macrophages and generates giant cells similar to those observed in granulomas. These results suggest that the small PV phenotype observed with *L*. *amazonensis* could be caused by CD36-mediated phosphatidylserine recruitment. If true, we would expect the enrichment of phosphatidylserine on the PV in a CD36-dependent manner. However, PS was not observed associated with the PV of WT or CD36^-/-^ macrophages, as monitored by Annexin V staining (S5 Fig). These results do not support the model of CD36-mediated recruitment of PS to the PV membrane.

### *CD36* ^-/-^ PVs have a normal acidification process

**Fig 10:**
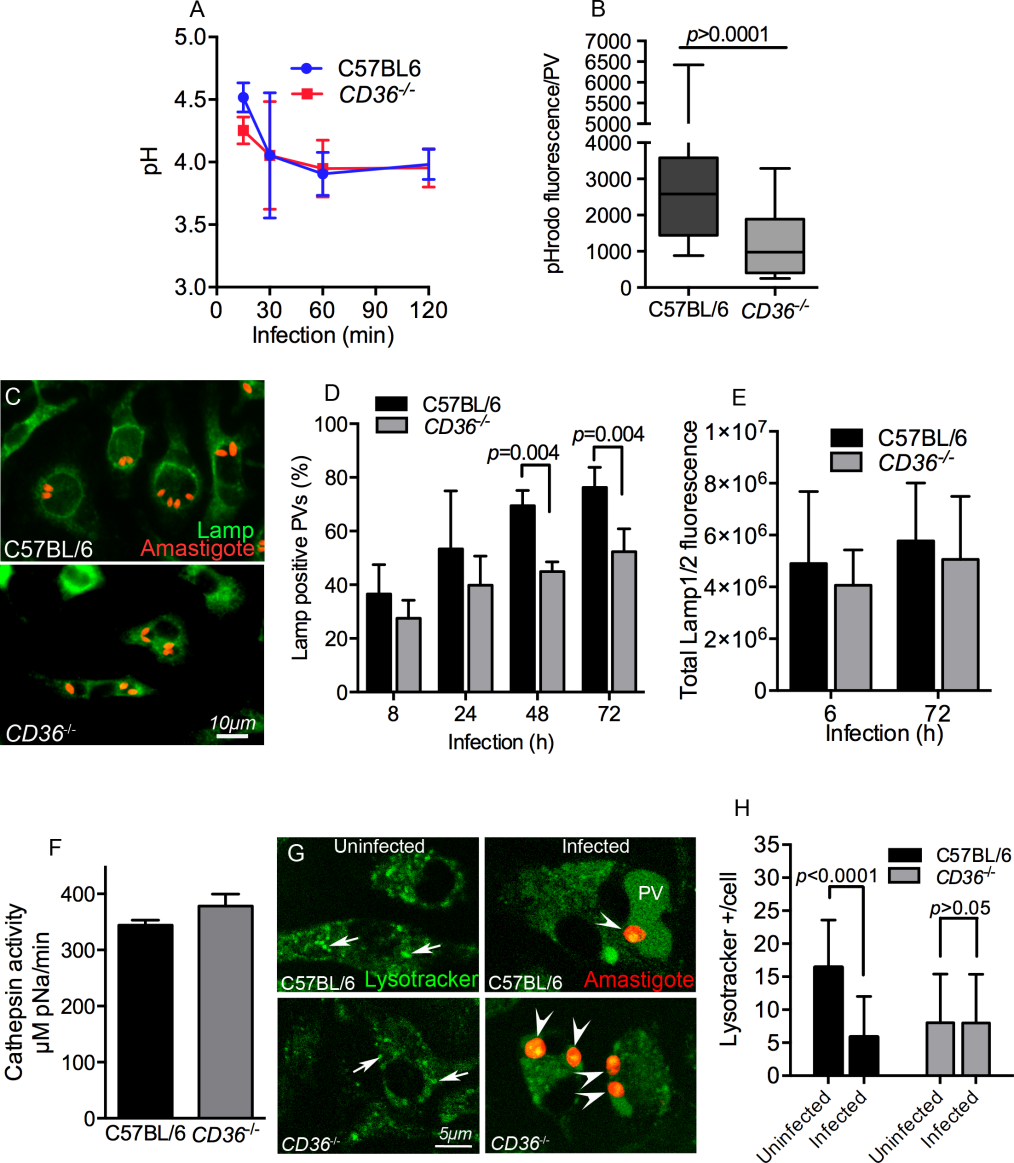
*CD36* ^-/-^ BMDMs have normal PV acidification and reduced lysosomal fusion to the PVs. (A) Acidification of PV was normal in *CD36* ^-/-^ macrophages. The pH was about 4.5 at 15 min of infection and stabilized at pH 4.0 at 1 h of infection in WT and CD36-deficient cells. The results shown are an average of 3 independent experiments. (B) Twenty four hours post-infection macrophages were loaded with a 15 min pulse of pHrodo Dextran and the accumulation of this tracer in each PV was measured by confocal microscopy after a 15 min chase. CD36-deficient PVs exhibited reduced accumulation of pHrodo coming from the endocytic pathway (t-test, n = 100). (C-E) Macrophages were infected and immunostained with anti-LAMP1/2 antibodies, followed by fluorescence microscopy imaging and quantitation. (D) C57BL/6 and *CD36* ^-/-^ macrophages had comparable percentages of LAMP positive PVs at early time points, but LAMP proteins gradually accumulated significantly more in the PVs from WT BMDMs at 48 h and 72 h post-infection. (E) The total fluorescence of LAMP1/2 is comparable between WT and CD36-deficient macrophages. Mean data of 300 cells imaged in 2 independent experiments. (F) To assess lysosomal protease activity, Cathepsin B activity was determined by measuring the hydrolysis of the synthetic substrate Z-RR-pNA. Lysosomal protease activity was normal in CD36-deficient macrophages indicating that CD36-deficient cells are not defective in lysosome biogenesis. (G-H) Macrophages were infected for 24 h and stained with Lysotracker. (G) Uninfected and 24 hour infected macrophages showing lysotracker signal (green), the arrows indicate typical Lysotracker positive organelles scored in the assay and the arrow heads indicate the amastigotes. (H) Lysotracker positive organelles were quantified in complete Z-stacks. WT cells lose lysosomes as they fuse with PV after parasite infection, while uninfected *CD36* ^-/-^ macrophages exhibited lower Lysotracker positive organelles which remain unaltered after infection. Results from a pool of 2 independent experiments with at least 27 cells in total, t-test.

To study the maturation of the PV, which is an altered phagosome, we next characterized the acidification of PV in WT and *CD36* ^-/-^ macrophages. We monitored the acidification of PV during the first 2 h post-infection using live *L*. *amazonensis* amastigotes that were uniformly immunolabeled with antibodies conjugated to Oregon Green 488 (pH-sensitive) or Alexafluor 647 (pH-stable). The fluorescence intensity of the infected macrophages was determined by flow cytometry. As shown in Fig 10A, *CD36* ^-/-^ and WT BMDMs similarly acidify the PV, which reaches a pH of 4.5 within 15 min post-infection and gradually stabilizes by 1 hour post-infection to an approximate pH of 4.0. Note, the opsonized parasites probably engaged the macrophage Fc receptors during internalization, however, this interaction did not interfere with the small PV phenotype of *CD36* ^-/-^ macrophages (S3 Fig).

### Reduced fusion of lysosomes/late endosomes to PV in *CD36* ^-/-^ macrophages

As PV acidification appeared normal in CD36-deficient cells, we also characterized endosome to PV fusion in *Leishmania*-infected BMDMs. Twenty four hours post-infection, macrophages were loaded with pHrodo Dextran and the amount of dye that accumulated in the PV was measured 30 min later. Notably, the total pHrodo fluorescence intensity per PV was higher in WT than in CD36-deficient macrophages, indicating that CD36 contributes to the fusion of PV with endocytic vesicles (Fig 10B).

This assay, however, does not discriminate which endocytic organelle delivers pHrodo Dextran to PVs. In particular, lysosomes are well known as an important source of material for PV enlargement [22, 24, 30, 31, 41]. Therefore, we hypothesized that the underdevelopment of PVs in *CD36* ^-/-^ macrophages could be caused by a dysfunction of lysosome biogenesis and/or fusion to the PV. To assess this, we measured the accumulation of the lysosomal markers LAMP1 and LAMP2 in the PV membrane of infected macrophages by immunostaining. Fluorescence microscopy imaging of WT macrophages showed that ~40% of PVs stained strongly for LAMP1/2 at 8 h post-infection, and this increased to ~80% by 72 h (Fig 10C and 10D). In *CD36* ^*-/*-^ macrophages, the percentage of LAMP positive PVs was similar to WT at earlier time points (8 h and 24 h), whereas there was significantly lower association of LAMP proteins with PV by 48 h and 72 h post-infection (Fig 10C and 10D).

The lower concentration of LAMP1 and LAMP2 in the *CD36* ^-/-^ PVs could be caused by lower quantity and/or reduced fusogenicity of lysosomes. To investigate this possibility further, two approaches were used to quantify the lysosomal content. The quantification of total fluorescence intensity of LAMP1 and LAMP2 in the macrophages, and the measurement of the activity of the lysosomal protease cathepsin B in total lysates. On average, the total fluorescence from LAMP1 and LAMP2 staining was similar in *CD36* ^*-/*-^ and WT macrophages (Fig 10E). Also, the cathepsin B activity was similar in lysates of uninfected *CD36* ^-/-^ and WT macrophages (Fig 10F). Together, these assays indicate that lysosomal biogenesis was normal in CD36-deficient macrophages, and therefore the lower rate of lysosomal fusion is the most likely explanation for reduced LAMP staining and small PV size.

In order to reinforce these findings, WT and *CD36* ^*-/*-^ BMDMs were stained with the lysotropic dye Lysotracker prior to and 24 h after infection. Cells were imaged by confocal microscopy and highly fluorescent organelles were scored through a complete Z-stack (Fig 10G). As expected, the number of Lysotracker-positive organelles in WT macrophages decreased 24 h post-infection, indicative of lysosomal fusion to PVs. However, the number of these organelles did not change in infected *CD36* ^-/-^ macrophages (Fig 10H) indicating a defect in lysosomal-PV fusion in the absence of CD36. While the CD36-deficient cells also showed a reduced overall level of lysotracker-positive vesicles, and this may contribute to the defect in PV enlargement, these vesicles were still detectable yet did not reduce after infection, as observed in WT macrophages. This strongly suggests that lower fusogenicity of late endolysosomal vesicles is the main cause of PV maturation dysfunction in the absence of CD36. In fact, this reduced fusogenicity may contribute to the overall reduction in large endolysosomal compartments observed prior to infection.

## Discussion

In this work, we developed an *in vivo L*. *amazonensis* infection model using *Drosophila melanogaster* to exploit the genetic tools available in this system. Characterization of this *Drosophila* infection model revealed that phagocytosis plays a pivotal role in parasite resistance. The central role of phagocytic plasmatocytes in *Leishmania* defense enabled an *in vivo* screening approach, in which RNAi was expressed specifically in these macrophage-like cells to evaluate the role of different genes in cellular anti-parasitic defense. Using the *Drosophila* infection model in a small scale screen, we identified six scavenger receptors, from three different families, that are involved in the defense of flies against *Leishmania*, suggesting that mammalian scavenger receptors could be more important to *Leishmania* infection than previously realized. Importantly, three of the scavenger receptors identified in our *Drosophila* screen were CD36-like (also known as the SR-B family). These *Drosophila* CD36s are critical for efficient phagocytosis of parasites and to control the infection. One of these CD36-like receptors, Crq was first identified as a plasmatocyte phagocytic receptor for apoptotic cells [50, 51], and was later linked to *S*. *aureus* recognition, which led to the discovery that mammalian CD36 is the co-receptor for the presentation of bacterial lipopeptides to TLR 2/6 [46]. The other two CD36-like receptors identified in our screen are uncharacterized.

Our most striking finding was that in mammalian systems CD36 plays a key role in the innate immune response to *L*. *amazonensis* infection by promoting PV expansion and parasite proliferation. Interestingly, CD36 is intensely recruited to the macrophage membrane upon amastigote contact, and then internalized within the phagocytic cup. Within a few hours after entry, CD36 is clustered at the anchoring site of the parasite, suggesting that the recruitment and fusion of endosomal vesicles is induced by parasites at this site. In the absence of CD36, the enlarged PV, typical of *L*. *amazonensis* infection, does not form. Although macrophages do not generate massive NO production upon amastigote infection [52, 53] the local NO environment was enough to arrest parasite proliferation in the small PVs of *CD36* ^*-/*-^ macrophages, in agreement with previous work [41]. This implies that the toxicity of the local NO environment is diluted in large PVs, but high concentrations of NO produced by IFN-γ/LPS-activated macrophages is sufficient to kill parasites.

The formation of similar clusters of CD36 upon contact with heat killed or fixed amastigotes indicates that CD36 aggregates upon binding to ligands found on the parasite surface, rather than through the action of some secreted virulence factors. Therefore, promastigotes probably do not recruit CD36 because they do not express CD36 ligands on their surface. Indeed promastigotes and amastigotes of *Leishmania* parasites have very different cell surface compositions. Promastigotes have a thick glycocalyx composed of lipophosphoglycan (LPG), proteophosphoglycan, gp63 and glycophosphatidylinositol lipids. In contrast, the amastigote glycocalyx has a low abundance of LPG and is rich in glycoinositolphospholipids (reviewed in [54]).

In agreement with the hypothesis of differential CD36 ligand exposure of promastigotes and amastigotes of *L*. *amazonensis*, the accumulation of CD36 following promastigote infection started to appear around 12 h post-infection, when amastigotes begin to form inside the PV. In this situation, the delay in CD36 accrual may be caused by expression of LPG and contribute to the delayed PV enlargement often observed in infections started with promastigotes [55, 56].

Collectively, the observation of the focal aggregation of CD36 in the large PV of *L*. *amazonensis* amastigotes, and not in the small PV of *L*. *amazonensis* promastigotes or *L*. *major* amastigotes is consistent with the finding that CD36 is essential for PV enlargement and maturation. Data presented here show that CD36 is critical for fusion of late endolysosomes with growing PVs. These data are in agreement with previous reports highlighting the importance of lysosomes for the enlargement of PVs observed in *L*. *mexicana* group infections [22, 24, 30, 31, 41].

While analysis of LAMP levels and cathepsin B activity were comparable between WT and *CD36* ^*-/*-^ macrophages, the total level of large lysotracker-positive organelles was decreased in CD36^-/-^ macrophages. This apparent discrepancy might be associated with the methodology, which considered only a fraction of total lysosomes, the ones with a strong and large Lysotracker signal; and therefore did not account for the numerous smaller and dimmer lysosomes [29]. This decrease in larger lysosomal vesicles suggests a fundamental defect in vesicle fusion in the absence of CD36.

Other members of the scavenger receptor B class have been linked to the maturation of phagolysosomes. The best studied example is the mammalian LIMP-2, which binds β-glucocerebrosidase at the endoplasmic reticulum and transports this enzyme into lysosomes [57, 58]. In addition, two other *Drosophila* Class B-related scavenger receptors, Crq and Debris buster (Dsb), were recently shown to be involved in the late stages of phagosome maturation necessary to degrade dendrite fragments phagocytosed by epithelial cells [59]. Thus, given the high degree of structural similarity amongst members of this class, the role of CD36 in maturation of PV is not unprecedented [60].

The underlying molecular mechanisms involved in the active fusion of PVs with endolysosomal vesicles and the link to CD36 requires further investigation. One possibility is that the CD36 aggregation observed on the *L*. *amazonensis* PV triggers CD36-signal transduction via receptor multimerization [61–64] or via interaction with other factors localized to the parasite anchoring site, such as MHC Class II, LAMP1 and LAMP2 [23, 26, 65]. Alternatively, CD36 may function as a channel to exchange lipids to and/or from the PV, including virulence factors that could increase fusogenicity of the PV. Such a channel has been demonstrated in the crystal structure solution of LIMP-2, and modeled in other members of the CD36 family [60]. For SR-BI this channel has been related to the bidirectional transport of cholesterol(esters) [66–68]. Another hypothesis is that CD36 on the PV membrane mediates the interaction with anionic lipids present in the incoming vesicle and promotes the fusion of these two organelles. Although we did not observe enrichment of phosphatidylserine in the PV, as suggested for the formation of giant granuloma cells [49], further studies will investigate if other lipids are involved in the CD36-dependent PV expansion.

It is important to emphasize that our work is focused on the study of amastigote forms and to further investigate the role of CD36 in the infection initiated by promastigotes, future studies should consider the infection of neutrophils, which are the primary cell type infected by *Leishmania* promastigotes during natural infection by sandfly bites [3, 69]. The neutrophilic delivery of amastigotes to macrophages was shown to be essential to establish the infection of *L*. *major* in the ear pinna of mice following a sand fly bite. Although the role of neutrophils in *in vivo* infection with parasites of the *L*. *mexicana* group is not completely understood, one interesting connection is that macrophage CD36, a known receptor for apoptotic cells, could be used to phagocytose apoptotic infected neutrophils, enabling a Trojan horse infection.

In conclusion, here we described the development of a novel *Drosophila* infection model of *Leishmania* which can be used to efficiently explore the innate immune pathways involved in interaction between phagocytes and *Leishmania* parasites. Studies in this model demonstrated that CD36-like receptors are essential for the fly to defend against *Leishmania* infection. In mammalian macrophages, we showed that *L*. *amazonensis* parasites exploit host CD36 function to favor their intracellular survival: amastigotes expose CD36 ligands to stimulate fusion with endolysosomal vesicles and promote PV enlargement, thereby reducing NO levels in the local environment and enhancing replication. On the other hand, promastigotes do not expose CD36 ligands and PV enlargement is delayed until these parasites differentiate into the intracellular adapted form, the amastigote. These findings open a new avenue of investigation to study the CD36 signaling pathways and cell biology involved in vesicular trafficking, phagosome maturation and host defense in leishmaniasis as well as in other diseases.

## Materials and Methods

### Ethics statement

All experiments were conducted according to the guidelines of the American Association for Laboratory Animal Science and approved by the Institutional Animal Care and Use Committee at the University of Massachusetts Medical School (Docket#: A-2056-13).

### Mice

*CD36* mutant mice were generated by targeted gene disruption in embryonic stem cells [70]. C57BL/6 and Balb/c mice were obtained from The Jackson Laboratory. All animals used in this work were 7 to 12-weeks-old and were maintained under pathogen-free conditions at the University of Massachusetts Medical School animal facilities.

### Bone marrow derived and immortalized macrophages

Femurs and tibia were dissected from mice and bone marrow was flushed with PBS using a 30G needle connected to a 10 mL syringe. The cells were cultivated in RPMI supplemented with 30% L929 cell-conditioned medium and 20% FBS and maintained in bacteriological petri dishes at 37°C and 5% CO_2_ [71]. The cultures were fed on day 3 and used on day 7. Immortalized macrophages were generated using J2 recombinant retrovirus carrying v-myc and v-raf oncogenes as described in Halle [72]. *MyD88* ^*-/*-^ and *TRIF* ^*-/*-^ immortalized macrophages were kindly provided by Dr Egil Lien (University of Massachusetts) and were generated from mutant mice BMDMs [73, 74].

### *Leishmania* parasites

GFP-transfected *L*. *amazonensis* (MHOM/BR/1973/M2269), and dsRed-transfected (RAT/BA/LV78)[75] were generously donated by Dr. Silvia R. Uliana (ICB-USP, Brazil) and Dr. Kwang-Poo Chang (Rosalind Franklin University of Medicine and Science), respectively. *Leishmania major* was of the MHOM/IL8/Friedlin strain. Promastigotes were cultivated *in vitro* in M199 medium supplemented with 10% FBS and 30 μg/mL Hygromycin B (GFP transfected) or 5 μg/mL of Tunicamycin (dsRed transfected) at 26°C.

### Intracellular amastigotes harvested from macrophages

Promastigote forms were differentiated axenically to amastigote forms by transferring the parasites to 199 media supplemented with 0.25% glucose, 0.5% trypticase, 40 mM sodium succinate (pH 5.4) and 20% FBS, at 1x10ˆ7 cells/mL at 32°C for 3 days. Axenic amastigotes were then used to infect Balb/c BMDM cultivated in 175 cm^2^ T-flasks (1.5x10ˆ7 cells/flask) at a multiplicity of infection of 10 parasites/BMDM. One week after infection cells were harvested from T-flasks and BMDMs were disrupted in PBS at 4°C using a glass dounce tissue grinder with teflon rod followed by two centrifugations at 210*g* for 8 min to remove intact BMDMs and cell debris, and one at 675*g* for 12 min to harvest the amastigotes.

### Production of retroviral vectors and transduction of immortalized mouse macrophages

The full length mouse CD36 cDNA (NM_007643.4) and mCherry were inserted in the retroviral transfer plasmid pCX4 Puro. 293T cells were transfected with the pCX4 mCherry-CD36, MLV gag-pol, and VSVG using GeneJuice transfection reagent (EMD Millipore) following the manufacturer’s recommendations. For the viral production, supernatants were refreshed 24 h and collected 48 h after transfection, filtered through a 0.45 μm pore filter and stored at -80°C until use. Immortalized macrophages were cultured in the presence of virus particles and 8 μg/ml Polybrene for 24 h, and then the transduced cells were selected with 3 μg/ml of puromycin for 4 days.

### Fly strains and infection assays

Fly lines used in this work are listed in the S1 Table. *Hml*(Δ)GAL4 was used as a hemocyte-specific driver, expressed from the 1st instar larvae [40, 76]. *Phago* ^*less*^ flies were generated by crossing virgin *hml*(Δ)-GAL4,UAS-eGFP female flies [31] with UAS-*bax*/CyO-actin-eGFP males. The progeny were maintained at 29°C after eclosion to optimize the function of the UAS/Gal4 system. Seven to 10 day old male flies were used in all experiments. A Nanoject (Drumond) equipped with a capillary needle was used for microinjections of 32 ƞL of a suspension of parasites and/or polystyrene beads in the abdomen. Polystyrene beads (0.2 μm diameter, FluoSpheres, Invitrogen F8805) were washed with PBS three times followed by centrifugation and suspended in PBS at concentration of 2% for injections. Forty thousand BMDM-derived amastigotes resuspended in PBS were injected per fly immediately after purification. We used at least 60 flies per sample for survival experiments that were monitored daily for at least 10 days. The survival curves of experimental flies were compared to flies injected with PBS and to control flies carrying the driver alone injected with the same preparation. The statistics of the survival curves were analyzed using Log-rank (Mantel-Cox) test. Susceptible flies were defined as those that reproducibly showed statistically significant decreases in survival with parasite but not with PBS injection.

### Measurement of parasite load in flies

The relative amount of parasites in adult flies was determined by limiting dilution. Infected flies were quickly washed in ethanol and individually ground with a tissue glass-teflon dounce homogenizer in 5 mL of Schneider media supplemented with 10% FBS, and 50 U/mL of a penicillin and streptomycin solution. The homogenates were filtered through a 70 μm mesh cell strainer to remove debris, and 100 μL of homogenate was serially diluted 24 times by a factor of 2 in triplicates. The cultures were analyzed for the presence of live promastigotes in the wells after 7 days of culture at 27°C, a technique modified from the limiting dilution method [77]. Alternatively, the flies were gently ground in 40 μL of Schneider media using a plastic pestle and the cell suspension loaded in an improved Neubauer chamber to count the dsRed parasites using CellProfiler cell image analysis software [78]. The same sample was used to determine the percentage of promastigotes in the flies by visual inspection.

### Confocal microscopy of hemocytes and parasite load determination

*hml*(Δ)Gal4-GFP adult flies expressing GFP in hemocytes were injected with parasites and/or beads and dissected in 20 μL of Schneider media on a glass slide on ice to drain the hemolymph. Samples from 5 flies were pooled in a coverglass bottom petri dish and centrifuged for 3 min at 200*g* to settle down the hemocytes in the bottom of the plate. The cells were visualized using a Leica SP8 AOBS laser-scanning microscope equipped with a 63X objective. The parasite load in hemocytes was determined in RNAi fly lines by counting the number of dsRed parasites in each GFP-expressing hemocyte using a fluorescence microscope. Each sample was a pool of 3 flies and 4 samples were scored per strain at 1 h post-infection.

### Macrophage infection and PV area measurement

Bone marrow derived macrophages were seeded (1x10ˆ5 cells/well) in 24 well plates containing round 12 mm glass coverslips and allowed to adhere overnight at 37°C and 5% CO_2_ in RPMI 1640 medium supplemented with 10% FBS and 5% L929 cell conditioned medium. Cultures were infected with amastigotes for 2 h and the coverslips were washed 3 times with RPMI media and transferred to a new plate. At the indicated times infected BMDMs were fixed with 4% paraformaldehyde for 15 min and mounted over a glass slide for microscopic imaging in a fluorescence microscope. The parasite load was determined by counting the intracellular GFP or dsRed-expressing amastigotes in each cell by microsocopy. The borders of PVs that contained GFP expressing parasites were delineated manually using ImageJ software to calculate the PV area.

293T cells were cultured in coverglass bottom 35mm dishes (Mattek Corporation), and transfected with mCerulean3-CD36-C-10, mCherry-CD36-C-10 and/or mCherry-Rab7A, gift from Michael Davidson, Addgene plasmid # 55405 [79], #55011, #55127 using GeneJuice (EMD Chemicals) according to the manufacturer’s recommendations. Cells were infected with amastigotes between 24 h and 48 h after transfection. For time-lapse live cell imaging, cells were infected for 24 h and incubated with pHrodo Red Dextran, 10,000 MW (Molecular Probes) for 15 min, washed and images were taken at intervals of 3 s at 34°C using Leica SP8 AOBS laser-scanning microscope equipped with a 63X oil immersion objective. The imaging conditions were optimized to use the minimum laser power to reduce phototoxicity.

### Parasite burden determination in BMDMs infected with *L*. *major*

To determine the parasite load of *L*. *major* in macrophages, the cultures were grown in 96 well plates, infected with metacyclic promastigotes for 3 h, then extensively washed to remove free parasites and cultured at 34°C. At indicated times post infection the media was removed and switched to Schneider media containing 10% FBS and cultured for 6 days at 25°C. These conditions induce macrophage death and release of parasites that proliferate in promastigote forms. At the end of 6 days in culture the promastigotes were counted using a hemacytometer to estimate the relative parasite load in each sample.

### Macrophage activation and NOS inhibition

Cells were cultivated in coverslips and nitric oxide synthase was inhibited by addition of N-nitro-L-arginine methyl ester (L-NAME) at 100 μM in the culture media for 1 h and washed prior to infection. For parasite killing assays, bone marrow derived macrophages were infected for 24 h and activated with 150 U/mL of IFNγ (eBioscience) and 50 ng/ml of ultra pure LPS (Invivogen) in the culture media. Infection was measured by counting the intracellular parasites and the percentage of infected cells.

### pHrodo Dextran endocytosis and transfer to PV

The fusion of endocytic vesicles with PV in infected macrophages was measured using a fluorescence-labeled dextran dye (pHrodo red dextran, MW 10000, Molecular Probes). This marker enters cells through endocytosis and increases fluorescence in low pH compartments like late endosomes and endosomes. Twenty four hours after infection, BMDM cultures were incubated with 50 μg/mL of pHrodo dye for 15 min, washed with PBS and incubated for 15 min in 20 mM Hepes buffer [pH = 7.4] containing 140 mM NaCl, 2.5 mM KCl, 1.8 mM CaCl_2_, and 1 mM MgCl_2_. The cultures were washed and the cells were imaged in a confocal microscope at the level of the parasite center for the next 20 min. The area of PV of the images was delineated manually and the intensity of fluorescence was measured using ImageJ.

### Determination of the pH of parasitophorous vacuoles from BMDMs

The pH of PVs was determined by dual fluorescence flow cytometry [80]. For the dual fluorescence staining, amastigotes were incubated on ice for 30 min with Balb/c infected mouse serum (1:1000), washed once and immunostained with the secondary antibodies conjugated to Alexafluor 647 or Oregon green 488 (Invitrogen) for another 30 min on ice and then washed once before using for infections. BMDMs were seeded in non-tissue culture treated 24 well plates one day before infection. The cultures were incubated on ice for 10 min to inhibit phagocytosis, and then the labeled parasites were added at an MOI = 4 and centrifuged 300*g* for 3 min to increase interactions between the BMDM and parasites. The plates were immediately incubated at 37°C in a water bath for 15, 30, 60 or 120 min. To detach the cells from the plate, the media was replaced by calcium and magnesium free PBS, kept on ice for 10 min and pipetted up and down until most of the cells were in suspension. Cell fluorescence was measured by flow cytometry and the pH was determined by the ratio of fluorescence of Oregon Green 488 to Alexafluor 647. Standard curves for each sample were prepared by equilibrating the cells in solutions with defined pH, 80mM potassium chloride, 30mM sodium chloride, 30 mM potassium phosphate, 5.5 mM glucose, 0.8 mM magnesium sulfate,1.3 mM calcium chloride, and 20 μM Nigericin to equilibrate the pH of the phagosomes to the pH of the media [81].

### LAMP1 and LAMP2 staining and quantification

Infected BMDMs were prepared as described above, and after fixation the cells were permeabilized with 0.05% Triton X-100 in PBS for 10 min, blocked with PBS 1% bovine serum albumin and stained with the monoclonal antibodies 1D4B (LAMP-1) and ABL-93 (LAMP2) from developmental studies hybridoma Bank (Iowa City, IA) overnight at 4°C followed by secondary antibody conjugated to Alexafluor 488 (Invitrogen) for 1 h at 37°C. The coverslips were washed and mounted with 50% glycerol on microscopic glass slides and imaged in a fluorescence microscope. For quantification assays we set the images to a high threshold and counted only PV that presented more than half of extension of the membrane enriched with LAMP1/2.

### Quantification of acidic compartments

Uninfected cells or cells infected for 24 h were stained with 100ηM Lysotracker for 2 h at 34°C. The images were taken in the continuous presence of Lysotraker using a Leica SP8 AOBS laser-scanning microscope equipped with a 63X objective, in z-stacks of 0.5 μm to cover the entire cells. The number of late endocytic compartments was determined using the 3D image counter plugin in ImageJ, and the counter was adjusted to consider only objects with intense fluorescence to avoid counting false objects from the background signal, and areas less than 8 μm^3^ to avoid counting PVs.

### Cathepsin B activity measurement

Cathepsin B activity assays were performed using the chromogenic substrate Z-RR-pNA (Enzo Life Sciences). 200 μM of substrate was diluted in 50 mM sodium acetate (pH = 5), 2.5 mM EDTA, and 0.1% Triton X-100. Cells were lysed in the assay buffer plus 1μM pepstatin A, 0.75μM aprotinin and 1mM PMSF (2x10ˆ7 cells/ml), and 10 μL of lysate was used per reaction in triplicate. The cleavage of substrate was monitored at 410 nm for 45 min at 15 min intervals and the amount of pNA released by Cathepsin B activity was determined using a pNA standard curve.

### RNA and qRT–PCR

Total RNA from BMDMs was isolated using the TRIzol reagent (Invitrogen) following the manufacturer’s recommendations. The RNA was then treated with DNase and re-extracted by phenol:chloroform method. cDNA was synthesized using iScript cDNA synthesis kit (BioRad) and quantitative PCR analysis was performed using SYBR Green (BioRad). The specificity of amplification was assessed for each sample by melting curve analysis and relative quantification was performed using a standard curve with dilutions of a standard. Oligonucleotide primers 5’-AGCAGAAGGTGATAGACCAGAA-3’ and 5’CCCACACTTGGATCATCAATGC-3’ were used to amplify the *Beige/LYST* and 5’-TCAGTCAACGGGGGACATAAA-3’ and 5’-GGGGCTGTACTGCTTAACCAG-3’ to amplify the loading control standard cDNA HPRT1 [41].

### Statistical analysis

The statistical analyses were performed using GraphPad Prism version 6.00 for Mac OS X, GraphPad Software, La Jolla California USA, www.graphpad.com. The tests and the criteria used for each comparison are reported in the Figure legends.

### Quantification and cellular localization of lipids

To quantify the incorporation of lipids, C57BL/6 and *CD36* ^*-/*-^ macrophages were cultured for 2 h in the presence of the fluorescent fatty acid and cholesterol analogs BODIPY FL C12 and CholEsteryl BODIPY 542/563 C11 (Molecular Probes) (5 μM final concentration). The cells were harvested from the culture dishes and the incorporation of the probes was measured by flow cytometry. The localization of the lipids was analyzed by confocal microscopy in paraformaldehyde macrophages infected for 4 h and incubated for 2 h with the probes.

### Phosphatidylserine staining

Infected BMDMs were fixed with 4% formaldehyde in PBS, permeabilized with 0.05% Triton X-100 in PBS for 10 min, blocked with PBS 1% bovine serum albumin and stained with Annexin V FITC (Apoptosis detection kit, eBiosciences). The coverslips were washed and mounted with 50% glycerol on microscopic glass slides and imaged in a confocal microscope.

## Supporting Information

S1 Movie PV fuses with vesicles of the endocytic pathway. 293T cells overexpressing mCerulean3-CD36 (Blue) were infected with amastigotes (green) for 24h and then loaded with pHrodo Red Dextran 10,000 MW for 15 min. The time-lapse confocal microscopy was initiated immediately after removal of the tracer. Most vesicles stained with CD36 also carried fluorescent dextran that gradually fused with the PV increasing the signal of pHrodo Red Dextran in the vacuole.
(AVI)

S1 Fig CD36 deficiency does not affect the incorporation of fatty acids and cholesteryl by mouse macrophages. C57BL/6 and *CD36* ^*-/*-^ macrophages were cultured for 2 h in the presence of the fluorescent fatty acid analog BODIPY FL C12 or CholEsteryl BODIPY 542/563 C11 in the culture media. The cells were harvested from the culture dishes and the incorporation of the probes was measured by flow cytometry. The incorporation of both lipids by *CD36* ^*-/*-^ macrophages was similar to WT measured by flow cytometry (A and B). The localization of the probes was analyzed by confocal microscopy in macrophages infected for 4 h and incubated for 2 h with the lipid probes. The probes could be visualized incorporated by C57BL/6 and *CD36* ^*-/*-^ macrophages and in the intracellular parasites (C and D, arrows).
(TIF)

S2 Fig The effect of CD36 deficiency on the *L*. *amazonensis* PV formation is independent of expression of *Beige* and TLR signaling. (A) Bone marrow derived macrophages were infected with amastigotes and processed for qRT-PCR. The CD36 deficiency did not induce higher *Beige* expression indicating that the small PV of *CD36* ^*-/*-^ macrophages is not related to *Beige* overexpression. (B) Immortalized macrophages from *MyD88* single and *MyD88*/*TRIF* double knockout mice had comparable PV size to WT cells at 24 h post-infection (n = 300, whiskers represent 10%-90% interval, Mann-Whitney t-test). Representative results from 3 independent experiments.
(TIF)

S3 Fig Serum opsonized amastigotes do not enlarge the PVs in *CD36* ^*-/*-^ macrophages. Amastigotes were opsonized with infected mouse serum and used to infect BMDMs. At 24 h after infection, WT infected cells presented normal large PVs (arrows) in contrast to the small PVs observed in *CD36* ^*-/*-^ macrophages.
(TIF)

S4 Fig The PVs of *L*. *major* does not display mCherry-CD36 accumulation and amastigotes proliferate normally in *CD36* ^*-/*-^ BMDMs. (A-B) Mouse immortalized macrophages expressing mCherry-CD36 were infected with immunolabeled *L*. *major* amastigotes. The small PVs containing *L*. *major* amastigotes did not present any sign of CD36 accumulation at 6 h (A) or 24 h (B) after infection. At least 50 infected cells were analyzed. Bar: 5 μm. (C-D) *CD36* ^*-/*-^ BMDMs presented normal phagocytosis and *L*. *major* proliferation. BMDMs were infected with promastigotes of *L*. *major* and the parasite burden was quantified at the indicated time points. The number of recovered parasites was highest at 3 h and reduced drastically at 24 h, as expected for the efficient macrophage killing of non-infective parasitic forms. A noticeable parasite proliferation in tight fitting PVs occurred at 96 h post-infection in both WT and *CD36* ^*-/*-^.
(TIF)

S5 Fig Phosphatidylserine does not accumulate in the PV membrane. Infected macrophages were fixed at indicated times, permeabilized, and stained with Annexin V FITC to detect phosphatidylserine. Phosphatidylserine was observed concentrated in several vesicles and the nuclear envelope, but Annexin V staining of PV membranes was not observed.
(TIF)

S1 Table Fly lines used for screening for *Leishmania* infection factors. (PDF)
