# Supplementary figures and images for: Leishmania amazonensis Engages CD36 to Drive Parasitophorous Vacuole Maturation
Source: PLoS Pathog. 2016 Jun 9;12(6):e1005669. doi: 10.1371/journal.ppat.1005669 (PMC4900624; doi:10.1371/journal.ppat.1005669)

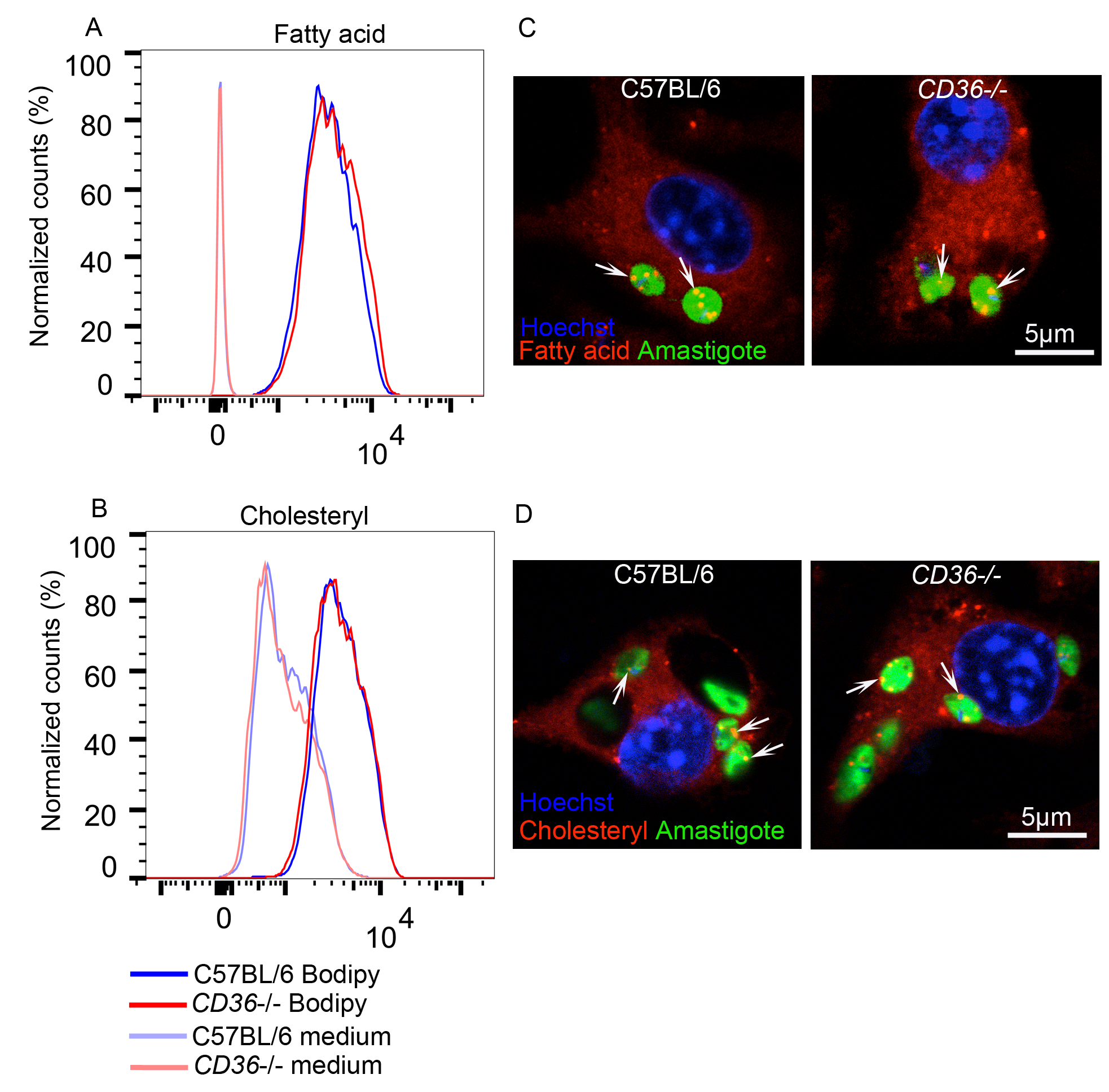

Supplement: S1 Fig — C57BL/6 and CD36 -/- macrophages were cultured for 2 h in the presence of the fluorescent fatty acid analog BODIPY FL C12 or CholEsteryl BODIPY 542/563 C11 in the culture media. The cells were harvested from the culture dishes and the incorporation of the probes was measured by flow cytometry. The incorporation of both lipids by CD36 -/- macrophages was similar to WT measured by flow cytometry (A and B). The localization of the probes was analyzed by confocal microscopy in macrophages infected for 4 h and incubated for 2 h with the lipid probes. The probes could be visualized incorporated by C57BL/6 and CD36 -/- macrophages and in the intracellular parasites (C and D, arrows). (TIF) [file ppat.1005669.s002.tif]

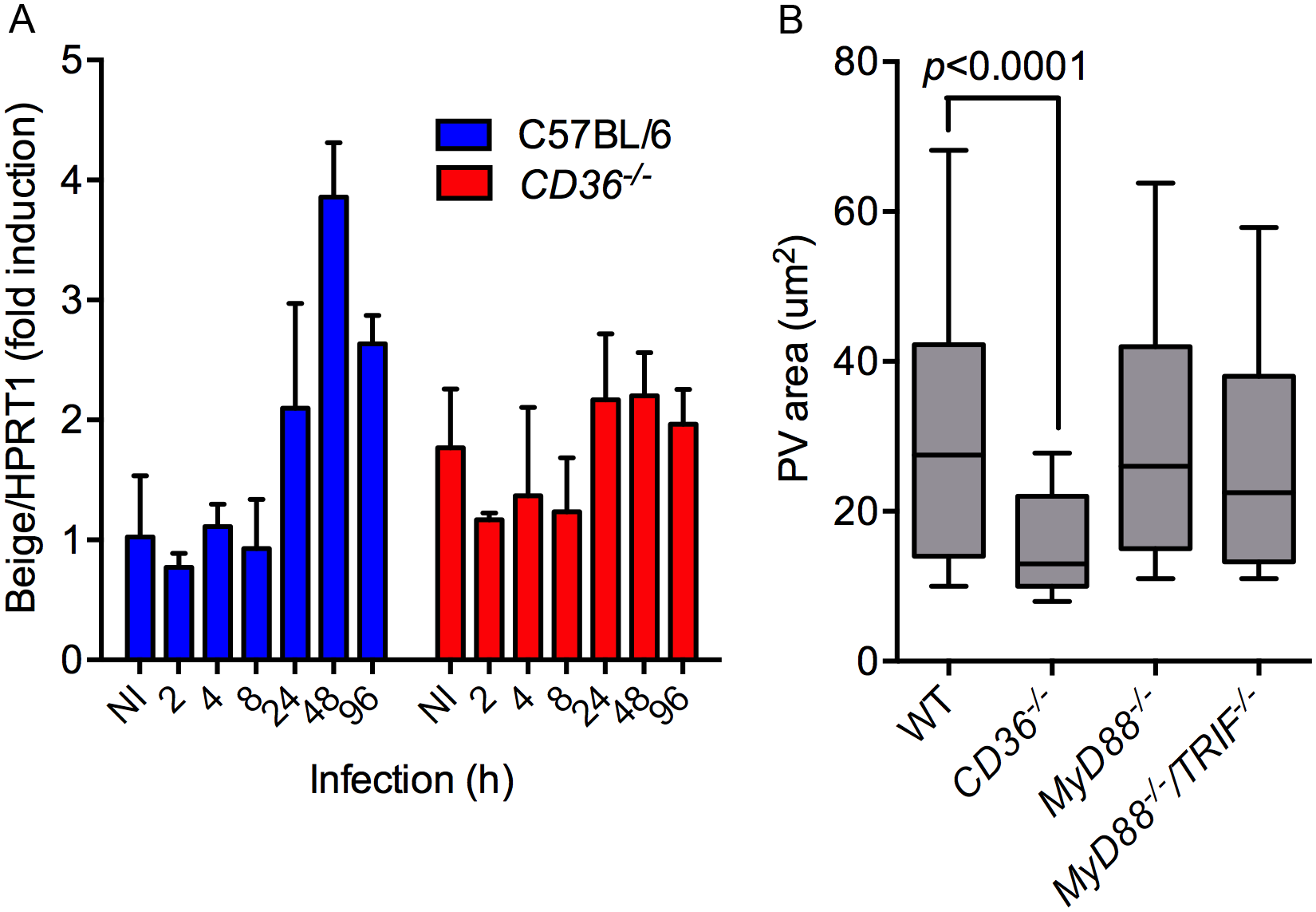

Supplement: S2 Fig — (A) Bone marrow derived macrophages were infected with amastigotes and processed for qRT-PCR. The CD36 deficiency did not induce higher Beige expression indicating that the small PV of CD36 -/- macrophages is not related to Beige overexpression. (B) Immortalized macrophages from MyD88 single and MyD88/TRIF double knockout mice had comparable PV size to WT cells at 24 h post-infection (n = 300, whiskers represent 10%-90% interval, Mann-Whitney t-test). Representative results from 3 independent experiments. (TIF) [file ppat.1005669.s003.tif]

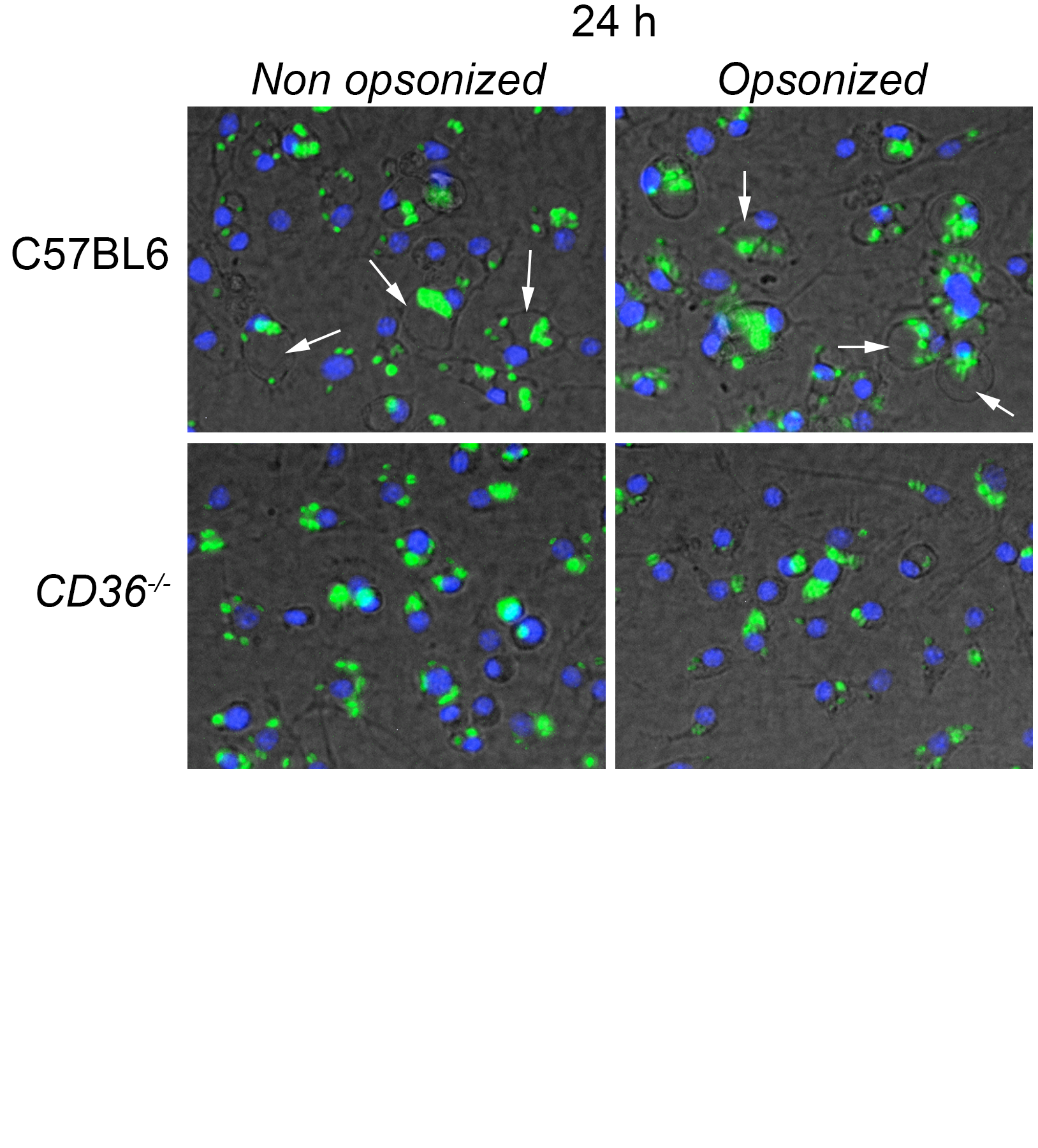

Supplement: S3 Fig — Amastigotes were opsonized with infected mouse serum and used to infect BMDMs. At 24 h after infection, WT infected cells presented normal large PVs (arrows) in contrast to the small PVs observed in CD36 -/- macrophages. (TIF) [file ppat.1005669.s004.tif]

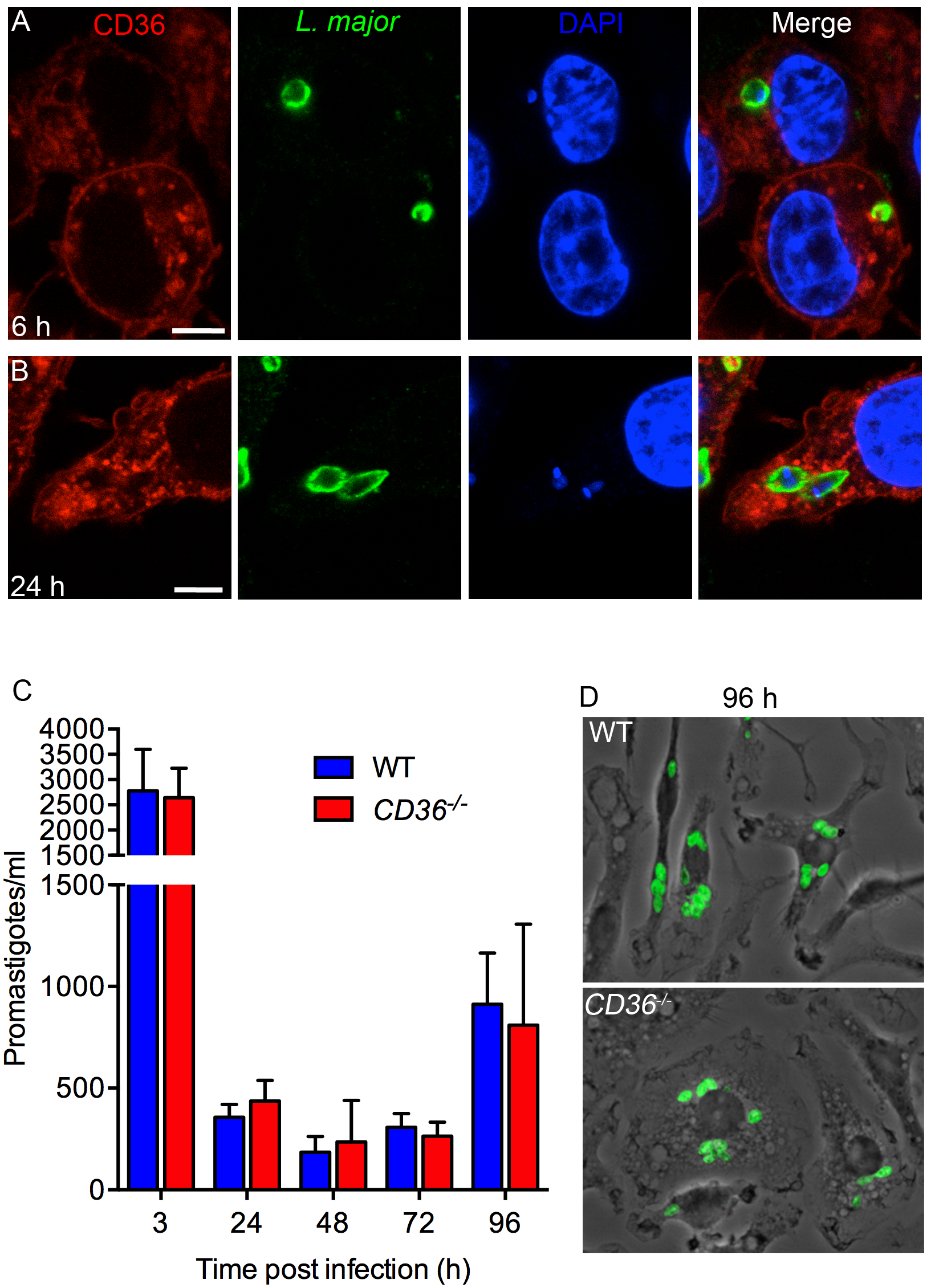

Supplement: S4 Fig — (A-B) Mouse immortalized macrophages expressing mCherry-CD36 were infected with immunolabeled L. major amastigotes. The small PVs containing L. major amastigotes did not present any sign of CD36 accumulation at 6 h (A) or 24 h (B) after infection. At least 50 infected cells were analyzed. Bar: 5 μm. (C-D) CD36 -/- BMDMs presented normal phagocytosis and L. major proliferation. BMDMs were infected with promastigotes of L. major and the parasite burden was quantified at the indicated time points. The number of recovered parasites was highest at 3 h and reduced drastically at 24 h, as expected for the efficient macrophage killing of non-infective parasitic forms. A noticeable parasite proliferation in tight fitting PVs occurred at 96 h post-infection in both WT and CD36 -/-. (TIF) [file ppat.1005669.s005.tif]

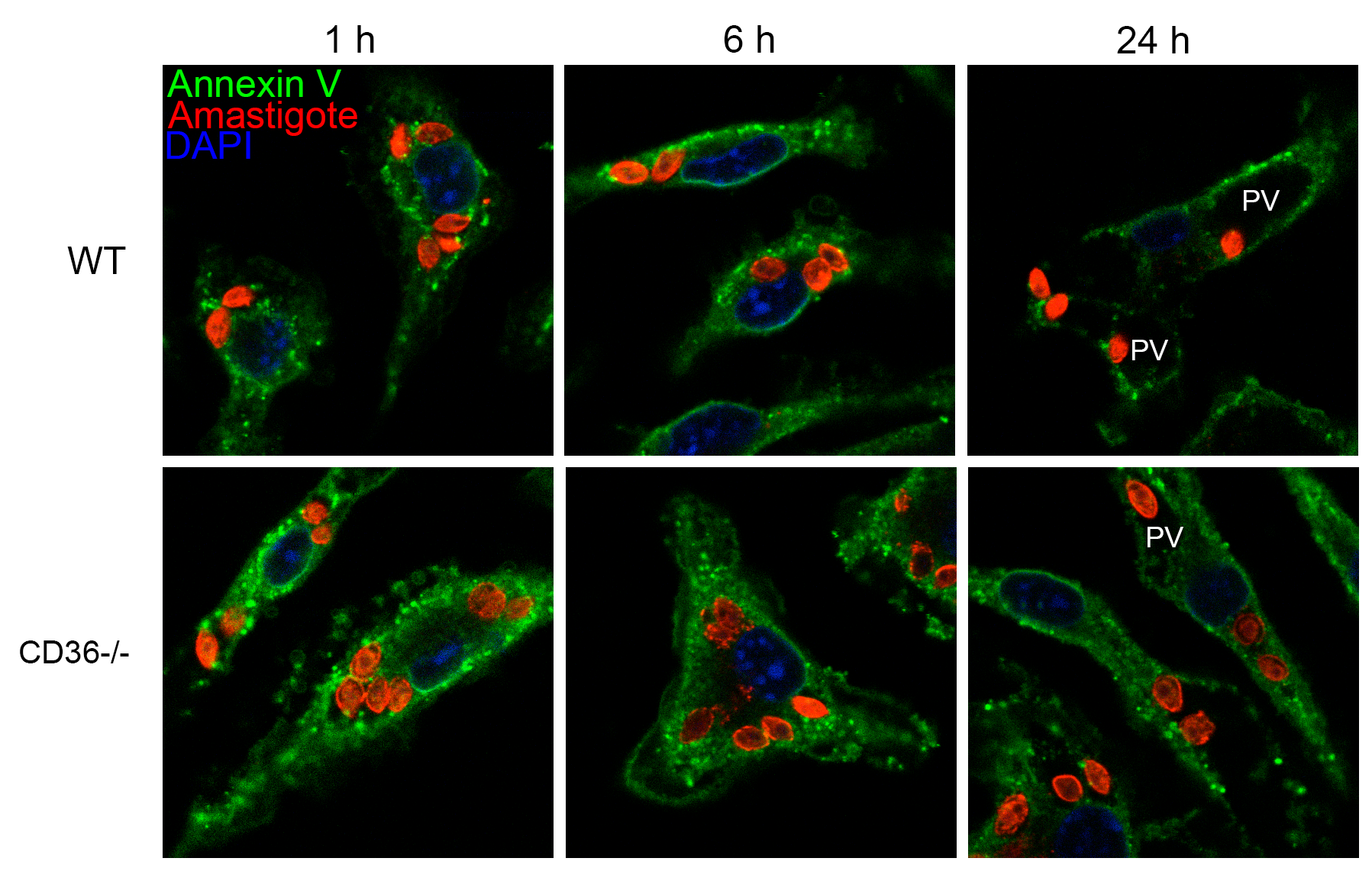

Supplement: S5 Fig — Infected macrophages were fixed at indicated times, permeabilized, and stained with Annexin V FITC to detect phosphatidylserine. Phosphatidylserine was observed concentrated in several vesicles and the nuclear envelope, but Annexin V staining of PV membranes was not observed. (TIF) [file ppat.1005669.s006.tif]
