# Supplementary material for: Leishmania amazonensis Engages CD36 to Drive Parasitophorous Vacuole Maturation
Source: PLoS Pathog. 2016 Jun 9;12(6):e1005669. doi: 10.1371/journal.ppat.1005669 (PMC4900624; doi:10.1371/journal.ppat.1005669)
Supplement: S1 Table — (PDF) [file ppat.1005669.s007.pdf]

S6 Table. Fly lines used in the screeening for factors related to *Leishmania* infection.

| Gene ID | Gene Name | Fly stock or genotype |
|---|---|---|
| CG4059 | ftz transcription factor 1 | VDRC v2959 |
| CG8556 | Rac2 | VDRC v50349 |
| CG6699 | β'-coatomer protein | VDRC v42071 |
| CG10345 | CG10345 | VDRC v51537/100252 |
| CG4280 | croquemort | VRDC v45883 |
| CG31741 | CG31741 | VDRC v7930 |
| CG31962 | Scavenger receptor class C, type III | VDRC v13032 |
| CG3212 | Scavenger receptor class C, type IV | VRDC v1330 |
| CG16880 | Nimrod C3 | VDRC v 22921 |
| CG1887 | debris buster | VRDC v4100 |
| CG2736 | CG2736 | VRDC v10272 |
| CG7228 | peste | VRDC v31155 |
| CG2727 | epithelial membrane protein | VRDC v12233 |
| CG7227 | CG7227 | VDRC v28612/108059 |
| CG12789 | santa-maria | VDRC v33153 |
| CG7000 | Sensory neuron membrane protein 1 | VDRC v42496 |
| CG3829 | CG3829 | VDRC v103492/103492 |
| CG8856 | Scavenger receptor class C, type II | VDRC v51504/100928 |
| CG42282 | Nimrod A | VDRC v39048 |
| CG33119 | Nimrod B1 | VDRC v19049 |
| CG31839 | Nimrod B2 | VDRC v105925 |
| CG16873 | Nimrod B5 | VRDC v15758 |
| CG8942 | Nimrod C1 | Trip 25787 |
| CG18146 | Nimrod C2 | VDRC v9976/Trip 25960 |
| CG16876 | Nimrod C4 | VDRC v36260 |
| CG6124 | eater | VDRC v4301/Trip 25863 |
| CG7447 | slowdown | VDRC v106464 |
| CG4845 | phagocyte signaling impaired | VDRC v21960 |
| CG4636 | SCAR | VRDC v21908/ |
| CG3664 | Rab5 | VRDC v103945 |
| CG2086 | draper | w;sp/cyo;*drpr*Δ5(tcc9)/Tm6,sd,Tb,c |
| CG4280 | croquemort | *crq*$^{-/-}$ |
| | | w; UAS-*Bax*/Cyo-Act GFP |
| | | w; *hml*ΔGAL4, UAS-GFP |
